# A beginner’s guide into curated analyses of open access datasets for biomarker discovery in neurodegeneration

**DOI:** 10.1038/s41597-023-02338-1

**Published:** 2023-07-06

**Authors:** Diana Gomes Moreira, Asad Jan

**Affiliations:** 1 Department of Clinical Medicine, Palle Juul-Jensens Boulevard 165, DK-8200 Aarhus N, Denmark; 2 Department of Biomedicine, Aarhus University, Høegh-Guldbergs Gade 10, DK-8000 Aarhus C, Denmark

**Keywords:** Neurodegenerative diseases, Biomarkers

## Abstract

The discovery of surrogate biomarkers reflecting neuronal dysfunction in neurodegenerative diseases (NDDs) remains an active area of research. To boost these efforts, we demonstrate the utility of publicly available datasets for probing the pathogenic relevance of candidate markers in NDDs. As a starting point, we introduce the readers to several open access resources, which contain gene expression profiles and proteomics datasets from patient studies in common NDDs, including proteomics analyses of cerebrospinal fluid (CSF). Then, we illustrate the method for curated gene expression analyses across select brain regions from four cohorts of Parkinson disease patients (and from one study in common NDDs), probing glutathione biogenesis, calcium signaling and autophagy. These data are complemented by findings of select markers in CSF-based studies in NDDs. Additionally, we enclose several annotated microarray studies, and summarize reports on CSF proteomics across the NDDs, which the readers can utilize for translational purposes. We anticipate that this “beginner’s guide” will benefit the research community in NDDs, and would serve as a useful educational tool.

## Introduction

Idiopathic Parkinson disease (PD) is a major neurodegenerative cause of motor disability in the ageing population worldwide, with characteristic loss of dopaminergic neurons in the midbrain *substantia nigra* (SN)-*pars compacta* and deposits of aggregated α-synuclein protein in the form of Lewy body (LB) pathology^1,2^. The pathological accumulation of aggregated α-synuclein in PD is not restricted to the SN, and is also found in several ‘extra-nigral’ locations in the brainstem (e.g,. DMX, the *dorsal motor nucleus of vagus* nerve and LC, *locus coeruleus*), and is hypothesized to underlie several motor and non-motor features of the disease^3–5^. An in-depth description of the etiological basis of clinical PD, including rare genetic underpinnings, can be consulted elsewhere^1,2^. Given the complex etiological basis of age-related neurodegeneration (e.g., idiopathic PD and late-onset Alzheimer disease- AD), it is plausible to postulate that neuronal dysfunction and demise result from interplay of several factors, including genetic and environmental influences, cellular adaptations to the deleterious effects of genetic variants that may increase disease risk, and  the extent of neuronal reserve for mitigating the metabolic challenges incurred by proteopathic stress^1,2,6^.

Therefore, there is significant interest both in the academia and in the industry sectors for the discovery and validation of candidate factors in biological fluids, which can be used as surrogate markers of neurodegeneration. The expectations are that: (i) the factor(s) confirms the presence of a disease (impact: diagnosis), (ii) may change over the course of the disease and/or in response treatment (impact: monitoring, stratification) and (iii) can be detected by readily accessible methods which do not require specialist training or infrastructure (impact: portability) (see an in-depth review on the subject matter elsewhere^7^). In this regard, considerable progress has been made for the biochemical and/or brain imaging-based detection of the classical markers of neuropathology (or their post-translationally modified forms such as phosphorylation), e.g., α-synuclein (gene symbol, SNCA) in PD and other synucleinopathies, β-amyloid in AD, tau in tauopathies (gene symbol, *MAPT*) or indicators of parenchymal damage (e.g., neurofilament ligh chain, gene symbol *NEFL*)^7^. The current stage of development and the predictive value of distinct ‘panels’ of pathological biomarkers is also available on the Alzforum website (Table 1). In addition, the discovery of biomarkers indicating early neuronal dysfunction and/or homeostatic response during disease progression is also gaining momentum, and several candidates are being investigated (for instance: neurogranin, neuron-specific enolase)^7^.

**Table 1:** Useful open access online portals.

Resource Identification	URL/Weblink	Remark
Gene Expression Omnibus (GEO)	https://www.ncbi.nlm.nih.gov/geo/	A database containing gene expression profiling and RNA methylation datasets managed by the NCBI
Alzforum Biomarkers	https://www.alzforum.org/alzbiomarker	The database on fluid biomarkers in AD and other neurodegenerative diseases
CSF Proteome Resource	https://proteomics.uib.no/csf-pr/	An online repository of mass spectrometry based proteomics experiments on human CSF, developed by University of Bergen (refs. ^12,13^)
Parkinson’s Progression Markers Initiative (PPMI)*	https://www.ppmi-info.org/	An open-access platform with datasets and biosample library for PD research, sponsored by the Michael J. Fox Foundation, USA
AD Knowledge Portal*	https://adknowledgeportal.synapse.org/	A public data repository that stores data and shares data analyses from studies in AD and dementia disorders. This large scale venture and analytical tools on the platform are funded by the National Institutes of Aging (NIA). Agora is an interactive visualization platform for assessing genetic association and differential gene/protein expression in AD
Also see: Agora	https://agora.adknowledgeportal.org/	
ALS-ST (Spatial Transcriptomics)	https://als-st.nygenome.org/	A suite of interactive visualization tools for exploring the ALS study by Maniatis, S. *et al*. (ref. ^96^)
Gene Set Enrichment Analysis (GSEA)	https://www.gsea-msigdb.org/gsea/	A joint project of UC San Diego and Broad Institute, GSEA contains curated gene sets and several analytical tools. GenePattern is an open-source platform for the analysis of gene expression (mRNA), genomics, proteomics and detailed network analyses
Also see: GSEA GenePattern	https://www.genepattern.org/	
Metascape	https://metascape.org/	A useful resource for obtaining gene annotation, functional enrichment and interactome analysis, from 40 independent knowledgebases within one integrated portal, developed by Zhou, Y. *et al*. (ref. ^97^)

*User registration is required to access the datasets from PPMI and AD Knowledge Portal.

This Resource article is intended primarily for the readers with strong interest in the discovery of pathogenic mechanisms and/or translational research in biomarkers for neurodegeneration in PD and related diseases. The basic framework is demonstrated by curated analyses of microarray datasets from human studies available in the Gene Expression Omnibus (GEO) repository of the National Center for Biotechnology Information (NCBI) (Table 1). We have primarily focused on the studies reporting gene expression profiling in patient-derived brain tissue specimen, since studies covering meta-analysis of blood samples^8^ or from animal studies are covered elsewhere^9^ (also see Discussion under Additional Resources).

Briefly, we performed curated gene expression profiling encompassing 3 canonical pathway gene sets derived from the KEGG pathway database available on the Gene Set Enrichment Analysis (GSEA) platform, namely: (i) glutathione metabolism, (ii) neuronal excitability and/or calcium signaling and (iii) regulation of autophagy. Although the existing literature points to significant perturbations in these pathways in neurodegeneration^10,11^, the choice of these panels is primarily to demonstrate the utility of the method described herein, without any *a priori* bias towards supporting or refuting a hypothesis. In other words, we do not intend to propose the select pathways as *bona fide* biomarkers, as compared with several established panels based on neuropathological association (i.e., α-synuclein, β-amyloid, tau). Instead, we mainly aim to provide information to the users on valuable resources, which could serve as compendia for assessing disease relevance of candidate markers (elaborated in Discussion). We complement these analyses of select markers with findings in human studies involving proteomics on the cerebrospinal fluid (CSF) by querying a unique online portal, The CSF proteome Resource developed by researchers at the University of Bergen, Norway (refs. ^12,13^ also see weblink to the portal in Table 1). Lastly, we provide annotated guides to several other microarray studies in NDDs, with focus on brain tissue, as well as include the salient findings in CSF-based studies (see Discussion under Additional Resources).

## Methods

### Gene expression analyses, using the NCBI GEO2R portal

Normalized gene expression data from the following trancriptomics datasets (with reference to the original study) was accessed on the NCBI GEO repository: (**1**) **GSE7621** (*substantia nigra-SN*; Controls, n = 9; PD, n = 16)- ref. ^14^, (**2**) **GSE43490** (*substantia nigra-SN, dorsal motor nucleus of vagus- DMX and locus coeruleus-LC*; Controls, n = 5–7; PD, n = 8)- ref. ^15^, (**3**) **GSE20146** (*globus pallidus, interna*-*GPi*; Controls, n = 10; PD, n = 10)- ref. ^16^ and (**4**) **GSE26927** (*substantia nigra-SN*; Controls, n = 7; PD, n = 12)- ref. ^17^. The dataset GSE26927 also contains the expression profiles in other common NDDs: Alzheimer disease (AD), Motor neurone disease (ALS) and Huntington disease (HD) (also Multiple sclerosis (MS), a demyelinating disease). Table S1 lists the details of the respective studies, including the control and case cohorts, microarray platforms and the brain regions analyzed.

### Curated analyses using GEO microarray datasets, step-by-step (see Figure S1)

1. Download the SUPPLEMENTARY EXCEL FILE 1 (.xlsx format) containing unique probe IDs for the platform GPL570 (for Dataset GSE7621 and Dataset GSE20146), GPL6104 (for Dataset GSE26927) and GPL6480 (for Dataset GSE43490) from the figshare repository (refer to the section: Data Availability). NOTE: The entries in the files have been arranged with gene symbols in alphabetical order.
2. Locate the unique probe IDs for the gene(s) of interest. For the current article, probe IDs are listed in Table S2. NOTE: The readers are encouraged to access the GSEA platform (https://www.gsea-msigdb.org/gsea/), where several curated gene lists from multiple resources (based on chemical and genetic perturbations or canonical pathways) are available.
3. On the NCBI GEO web portal (https://www.ncbi.nlm.nih.gov/geo/geo2r), enter the GEO accession for the desired dataset e.g. GSE7621 in the ‘**Search**’ area and click Search. This action will load the page summarizing the details regarding the particular study (also see, Figure S1).
4. NOTE: throughout this analysis, the same web interface will be active during all the steps below. For example, for the dataset GSE7621, all the following steps are carried out on: https://www.ncbi.nlm.nih.gov/geo/geo2r/?acc=GSE7621.
5. Scroll down to locate ‘**Analyze with GEO2R’**. A new page will load the sample accession IDs and other details (e.g., control or PD).
6. Locate ‘**Profile Graph’**, enter the unique probe ID for a gene of interest and hit **Set**. For example, the unique probe ID for the gene symbol *G6PD* in GPL570 is 202275_at. NOTE: This action will load the profile graph (bar chart) across all the samples with accession IDs on the *x-axis*.
7. Then click on ‘**Sample Values’** to get a pop-up display of all the data in the chart.
8. Copy all the content from this display and **paste as text** into an excel sheet (or similar data analyses interface).
9. Save the file and repeat the steps 1–8 for all the desired genes of interest.
10. Plot the data in the desired format and analyze the significance by the relevant statistical method.

NOTE 1: In this demonstration, the values for expression data for each probe (gene) have been normalized to the mean value of the control samples, such that control expression = 1 ± standard deviation. Pair-wise comparisons were performed by Mann-Whitney test using Graphpad Prism software. Also see Figure S1 for a pictoral overview of the steps 4–8.

NOTE 2: Additional considerations on the data analyses, especially multiple sampling and false discovery rate are briefly discussed under “*The methodological context*” in DISCUSSION.

### Differential analyses using GEO microarray datasets, step-by-step (see Figure S2)

1. It is also possible to perform global analyses (e.g. differential expression, control vs. PD etc.) using the built-in function of the GEO2R interface (https://www.ncbi.nlm.nih.gov/geo/geo2r) (**GEO2R/ Quick start)**, in contrast with curated expression analyses presented above. For this purpose, start with loading the GSE dataset, as outlined in steps 3–4 under curated analyes.
2. Then, **Define Groups** and assign samples to each group (eg, control and PD)
3. If need be modify the statistical parameters and desired visualization plots under **Options**.
4. Click **Analyze**, to obtain a downloadable table (list) ranked according to the significance and visualization plots (e.g. log(2) fold change, box plots, Mean-variance trend)
5. See Figure S2 for a pictoral overview of this method. We have also uploaded SUPPLEMENTARY EXCEL FILE 2 (.xlsx format) in the figshare repository (refer to the section: Data Availability), containing global expression profile of the top altered genes (i.e., differential expression between control and PD samples) in the PD datatsets GSE7621, GSE43490 and GSE20146.

### CSF proteomics portal, step-by-step (see Figure S3)

A detailed description of the interface with case studies is illustrated by Guldbrandsen *et al*.^12^.

1. Access the weblink https://proteomics.uib.no/csf-pr/ and click “**Search protein Data**”
2. In the Search box, enter the unique identifier (e.g., Uniprot ID), select the input type (e.g., “Protein Accession”) and the disease category (AD, PD, MS, ALS) and then click “**Search**”. NOTE: This will generate a graphical overview of the detected proteins along with the disease category
3. In the graphical overview, select a unique marker and/or disease category to view study details and click **Load**. Alternatively, simply click **Load** without selecting any marker to view all data on all the markers (and in disease categories entered at step 2)
4. Click on the **Protein Table icon** on the left side of the interface (illustrated in Figure S3), which will trigger a sub-Menu **Protein Details**
5. Click on the **Protein Details** to access the marker trend (ie., increased, decreased, equal/unaltered), with reference to the original research report and peptides detected.
6. Download the information by using the **Export Table** function
7. A pictoral overview is presented as Figure S3, including additional features in the interface (Disease comparisons, Protein Overview) which can be used to filter the information.

## Results

**Fig. 1:**
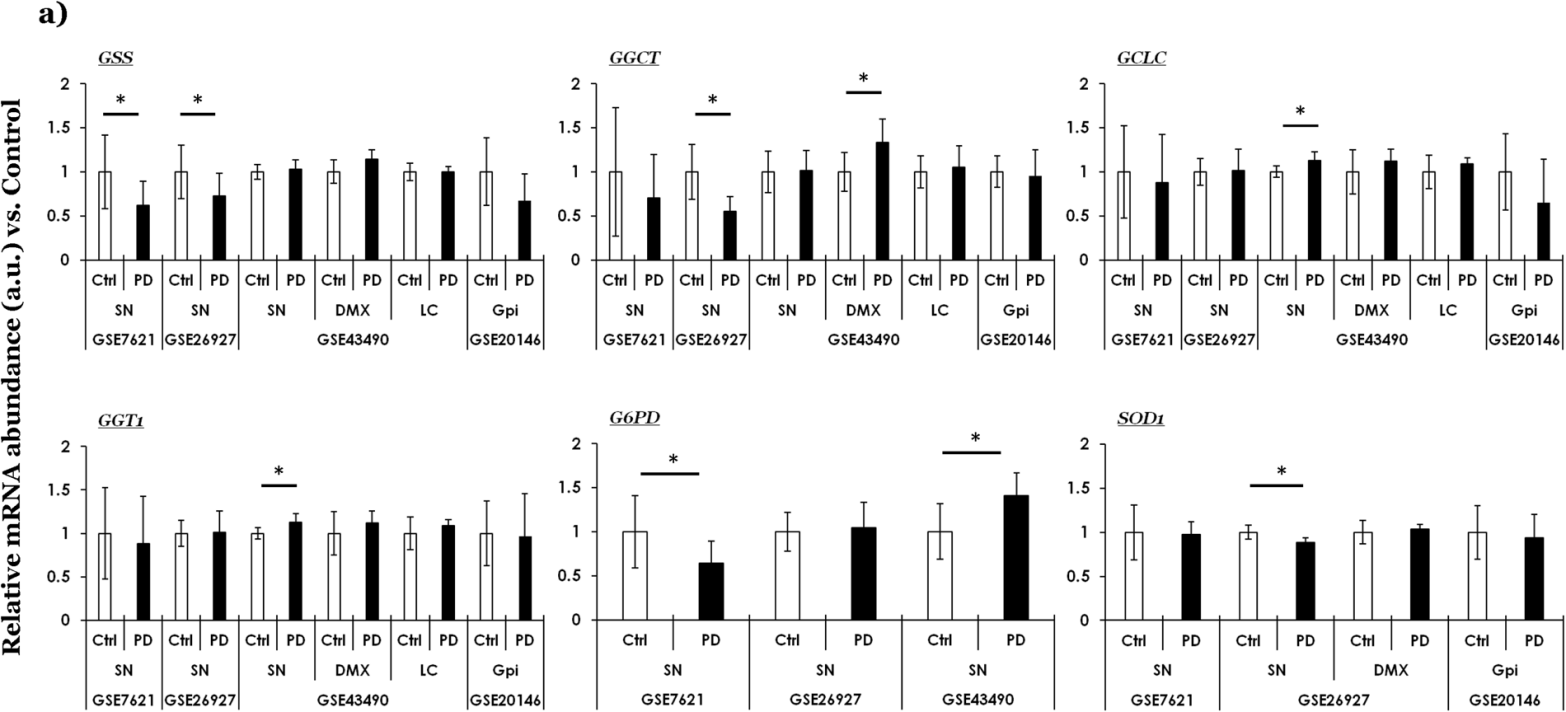
Curated gene expression analyses of glutathione biogenesis related factors within GEO microarray datasets in PD. (**a**) Glutathione synthetase (gene symbol, *GSS*), gamma-Glutamylcyclotransferase (gene symbol, *GGCT)*, gamma-glutamylcysteine synthetase (gene symbol, *GCLC)*, gamma-glutamyltransferase/transpeptidase 1 (gene symbol, *GGT1)*, glucose-6-phosphate dehydrogenase (gene symbol, *G6PD)* and superoxide dismutase (gene symbol *SOD1)*. The values across the datasets are expressed relative to the controls in each microarray dataset, i.e., mean value of control samples = 1 (a.u., arbitrary units). Error bars represent standard deviation of the mean, s.d. Pair-wise comparisons were assessed by Mann-Whitney test- only significant differences (*p ≤ 0.05, **p ≤ 0.01, ***p ≤ 0.005) are highlighted. The number of controls and cases, microarray platforms and  the references to original studies are included in Table S1. Unique probe IDs within each dataset are included in Table S2. Ctrl (controls); PD (Parkinson disease); SN (*substantia nigra*); DMX (*dorsal motor nucleus of vagus*), LC (*locus coeruleus)*; GPi (*globus pallidus interna*).

**Fig. 2:**
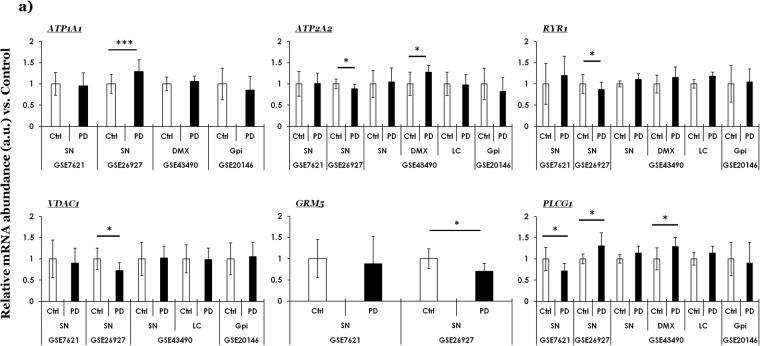
Curated gene expression analyses of neuronal excitability/calcium signaling related factors within GEO microarray datasets in PD. (**a**) Sodium-potassium ATPase, catalytic subunit alpha-1 (*ATP1A1*), sarcoplasmic/endoplasmic reticulum (ER) Ca^2+^ transporting ATPase 2, alias SERCA2 (*ATP2A2*), ryanodine receptor 1 (*RYR1*), voltage dependent anion channel 1 (*VDAC1*), glutamate metabotropic receptor 5 (*GRM5*), and phospholipase c gamma 1 (*PLGC1*). The values across the datasets are expressed relative to the controls in each microarray dataset, i.e., mean value of control samples = 1 (a.u., arbitrary units). Error bars represent standard deviation of the mean, s.d. Pair-wise comparisons were assessed by Mann-Whitney test- only significant differences (*p ≤ 0.05, **p ≤ 0.01, ***p ≤ 0.005) are highlighted. The number of controls and cases, microarray platforms and original studies are included in Table S1. Unique probe IDs within each dataset are included in Table S2. Ctrl (controls); PD (Parkinson disease); SN (*substantia nigra*); DMX (*dorsal motor nucleus of vagus*), LC (*locus coeruleus)*; GPi (*globus pallidus interna*).

**Fig. 3:**
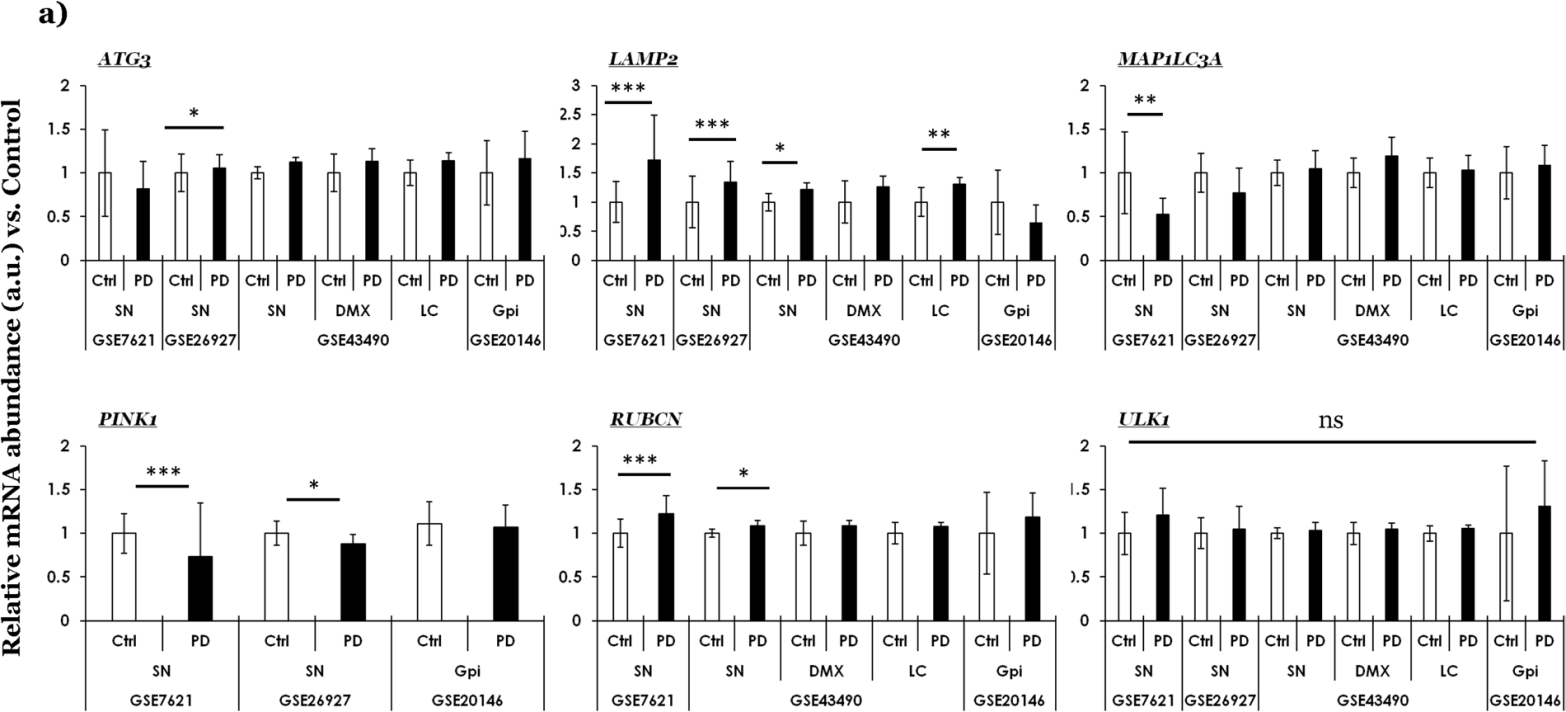
Curated gene expression analyses of factors involved in the regulation of autophagy within GEO microarray datasets in PD. (**a**) Autophagy Related 3 (*ATG3)*, lysosomal associated membrane protein 2 (*LAMP2)*, microtubule associated protein 1 Light Chain 3, alias LC3 alpha (*MAP1LC3*,) PTEN Induced Kinase 1 (*PINK1)*, rubicon autophagy regulator (*RUBCN*) and Unc-51 like autophagy activating kinase 1 (*ULK1*). The values across the datasets are expressed relative to the controls in each microarray dataset, i.e., mean value of control samples = 1 (a.u., arbitrary units). Error bars represent standard deviation of the mean, s.d. Pair-wise comparisons were assessed by Mann-Whitney test- only significant differences (*p ≤ 0.05, **p ≤ 0.01, ***p ≤ 0.005; ns = not significant) are highlighted. The number of controls and cases, microarray platforms and original studies are included in Table S1. Unique probe IDs within each dataset are included in Table S2. Ctrl (controls); PD (Parkinson disease); SN (*substantia nigra*); DMX (*dorsal motor nucleus of vagus*), LC (*locus coeruleus)*; GPi (*globus pallidus interna*).

As alluded above, the utility of using the NCBI GEO platform is demonstrated by probing the expression of select factors- in an unbiased manner- involved in glutathione metabolism, neuronal excitability and/or calcium signaling, and in the regulation of autophagy (Figs. 1–3). Moreover, analyses of the expression profile of select disease markers (e.g., *SNCA*) in PD datasets are included for the readers’ reference (Fig. S4a), as well as summary of  the findings in other diseases (i.e, AD, ALS, HD and MS) are presented throughout Figs. S5–S7. Important details regarding the identity of select markers, unique probe IDs and a brief note of their function is presented in Table S2.

### Glutathione (GSH) biogenesis

Glutathione (GSH) is a major cytoprotective molecule and serves as a co-factor in several enzymatic reactions concerned with maintaining intracellular redox homeostasis. GSH is synthesized from cysteine, glutamate and glycine by the action of glutathione synthetase (gene symbol, *GSS*), as elaborated elsewhere^18^. Several lines of evidence implicate perturbations in redox homeostasis in the pathogenesis of neurodegeneration in PD and related diseases^11,19,20^. Figure 1 shows the expression profile of select genes in PD, while Figure S5 shows the expression changes across AD, HD, ALS, MS and PD.

The data (Fig. 1a) show that the expression of *GSS* is significantly reduced in the PD SN within 2 datasets (GSE7621 and GSE26297), while is relatively unaltered in GSE43490. Moreover, the expression levels in DMX, LC and Gpi were not significantly altered compared to the controls in the 4 datasets examined. The expression profile of gamma-glutamylcyclotransferase (gene symbol, *GGCT*) shows reduction in PD SN (significant in GSE26297; not significant in GSE7621), and intriguingly is upregulated in the PD DMX (GSE43490). Within the same dataset (GSE43490), the expression of gamma-glutamylcysteine synthetase (gene symbol, *GCLC*) and gamma-glutamyltransferase/transpeptidase 1 (gene symbol, *GGT1*) is only significantly increased in the PD SN.

Apart from the anti-oxidant genes directly involved in GSH biogenesis/metabolism, two additional factors in the homeostatic maintenance of cellular redox balance are worthy of note (Fig. 1a). The first one is glucose-6-phosphate dehydrogenase (gene symbol, *G6PD*), with an interesting expression pattern in SN across 3 PD cohorts. Briefly, while *G6PD* expression levels show a reduction within the PD cohort belonging to GSE7621, its expression levels in the other datasets is either relatively unaltered (GSE26297), or show a significant increase (GSE43490). Lastly, the expression of superoxide dismutase (gene symbol, *SOD1*) only shows a slight but significant reduction in PD SN within the dataset GSE26297, and is relatively unaltered in GSE7621 or GSE20146. Regarding the expression profile of these genes in other diseases (Fig. S5a, GSE26927), notable findings include: HD (reduced levels of *GSS*, *GCLC* and *GGT1*) and ALS (increased levels of *G6PD* and reduced expression of *SOD1*). The findings in PD SN within the dataset GSE26927 are already presented in Fig. 1a, and included in Fig. S5a only for comparison.

### Neuronal excitability and/or calcium signaling

While the critical role of intracellular calcium in the maintenance of neuronal excitability applies to several neuronal populations, the midbrain dopaminergic neurons are particularly vulnerable to calcium dyshomeostasis, which in turn is linked to oxidative stress in neurons^21–23^. Figure 2 and Fig. S6 show the expression profile of select genes involved in neuronal excitability (Fig. 2a, PD; Fig. S6a, across other diseases). Significantly altered genes across datasets and their functions include: (i) Sodium-potassium ATPase, catalytic subunit alpha-1 (gene symbol, *ATP1A1*), (ii) Sarcoplasmic/endoplasmic reticulum (ER) Ca^2+^ transporting ATPase 2, alias SERCA2 (gene symbol, *ATP2A2*), (iii) Ryanodine receptor 1 (gene symbol, *RYR1*), (iv) Voltage dependent anion channel 1 (gene symbol, *VDAC1*), (v) Glutamate metabotropic receptor 5 (gene symbol, *GRM5*) and (vi) Phospholipase c gamma 1 (gene symbol, *PLGC1*).

The notable findings in PD datasets are briefly presented as follows: (a) increased expression levels in PD SN (Fig. 2a, within GSE26927: *ATP1A1* and *PLCG1*; but also note decreased expression of *PLCG1* within the dataset GSE7621), (b) decreased expression levels in PD SN (Fig. 2a, within GSE26927: *ATP2A2*; *RYR1*, *VDAC1*, *GRM5*) and (c) increased expression levels in PD DMX (Fig. 2a, in GSE43490: *ATP2A2* and *PLCG1*). Regarding the expression profile of these genes in other diseases (Fig. S6a; GSE26927), notable findings include: HD (increased level of *RYR1* and *PLCG1* and reduced levels of *VDAC1* and *GRM5*,). Also, the findings in PD SN within the dataset GSE26927 are already presented in Fig. 2a, and included in Fig. S6a only for comparison.

### Regulation of autophagy

Autophagy is the proteolytic degradation of damaged organelles and misfolded proteins, and plays a key role in the cellular energy homeostasis. The process is controlled by several key mediators, and dysregulated autophagy is implicated in the pathogenesis of several neurodegenerative diseases including PD (the reader is encouraged to consult in-depth review on the topic elsewhere^24^). Figure 3 and Fig. S7a show the expression profile of select genes involved in autophagy regulation (Fig. 3a,PD; Fig. S7a, across other diseases). Significantly altered genes across datasets and their functions include: (i) Autophagy Related 3 (gene symbol, *ATG3*), (ii) Lysosomal associated membrane protein 2 (gene symbol, *LAMP2*), (iii) Microtubule associated protein 1 Light Chain 3, alias LC3 alpha (gene symbol, *MAP1LC3*), (iv) PTEN Induced Kinase 1 (gene symbol, *PINK1*), (v) Rubicon autophagy regulator (gene symbol, *RUBCN*), (vi) Unc-51 like autophagy activating kinase 1 (gene symbol, *ULK1*) and (vi) TANK binding kinase 1 (gene symbol, *TBK1*).

The notable findings in PD datasets are briefly presented as follows: (a) increased expression levels in PD SN (Fig. 3a, within GSE26927: *ATG3*, *LAMP2* and *RUBCN;* within GSE7621 and GSE43490: *LAMP2* and *RUBCN*), (b) decreased expression levels in PD SN (Fig. 3a, within GSE26927: *MAP1LC3* and *PINK1*; also note decreased expression of *PINK1* within the dataset GSE7621) and (c) increased expression levels in PD LC (Fig. 3a, in GSE43490: *LAMP2*). Regarding the expression profile of these genes in other diseases (Fig. S7a; GSE26927), notable findings include: HD (reduced levels of *PINK1* and *ULK1*) and MS (slight but significantly reduced levels of *ATG3*). Also, the findings in PD SN within the dataset GSE26927 are already presented in Fig. 3a, and included in Fig. S7a only for comparison. Lastly, the expression values of *TBK1* in the PD datasets were not significant, while the probe IDs for *TBK1* and *RUBCN* were not found in GSE26927.

## Discussion

To demonstrate the utility of omics datasets available in the public domain for translational research in biomarker discovery in NDDs, we have presented examples from the NCBI GEO repository, with curated gene expression analyses of select biochemical pathways (Figs. 1–3; Figs. S5-7). The purpose is to familiarize the users with resources containing patient-derived data, which can be combined with other resources (e.g., proteomics datasets, metabolomics, imaging studies- Table 1; also see Additional Resources below) to build a refined picture of the mechanisms in neurodegeneration. We have previously used these resources to interrogate the disease relevance of candidate markers in AD and PD, and to establish their significance using a multi-pronged approach involving validation in post-mortem brain specimen and in cellular and animal models (elaborated below)^25–27^.

It is worthwhile to consider that for a high degree of confidence in a panel of candidate markers would necessitate the implementation of a multi-source standardized approach, since overreliance on one resource of data, or a single cohort of patients without longitudinal assessment, may be potentially misleading. For example, in the panel of markers assessed in this article, it is noteworthy that the expression profile of the genes examined from the microarray datasets was not uniformly altered across the PD cohorts (Figs. 1–3). This could potentially reflect heterogeneity in the cohorts (e.g., stage of pathology, extent of neurodegeneration, treatment regimen, co-morbidities etc.). Furthermore, reliance on a single marker can also present a confounding factor. For instance, while it can be noted that the expression of *LAMP2* and *RUBCN* is increased in the PD SN (Fig. 3a; GSE7621 and GSE43490), the expression pattern of *G6PD* (Fig. 1a) and *PLGC1* (Fig. 2a) shows inconsistency between the datasets. This limitation is also highlighted by variations in the expression of disease associated genes (e.g., *SNCA*) in the PD datasets (Fig. S4a).

One approach to address this issue could be to prioritize findings which are vetted through an *a priori* analytical plan incorporating additional factors, importantly the biological context and the methodological context. Accordingly, one could survey the literature and relevant resources (Table 1) for strength of the evidence regarding the association of a factor of interest to the disease category. For example, one could follow up to investigate if the factor(s) of interest has been detected in the context of neuropathology and/or is reported to be significantly altered in biological fluids, such as CSF and plasma. Furthermore, it is also imperative that enough statistical consideration is given to account for the confounding factors in biological datasets, such as the false discovery rate (FDR) and tendency for variation in samples.

### The biological context

To illustrate the first aspect (i.e., the biological context), we extended our approach to interrogate if the select markers (Table S2; Figs. 1–3): (i) have been reported to be associated with neuropathology in PD and/or (ii) have been detected in human CSF studies. Impaired redox homeostasis plays an important role in the pathogenesis of PD^28^, with SOD serving as a crucial anti-oxidant defense in the detoxification of intracellular superoxide free radicals^29^. In the CSF and brain tissue of PD patients, some reports show that SOD activity is considerably increased, thus potentially indicating tissue response to oxidative stress in PD brain^30–32^. Related to this, post-mortem studies show that the levels of GSH, another important anti-oxidant, are also decreased in the SN, putamen, globus pallidus, nucleus basalis of Meynert, amygdaloid nucleus, and frontal cortex in LB diseases including PD^33,34^. Similarly, one study showed that the GSH content in the hippocampus and cortex of PD patients was 40% lower when compared to the control specimen^35^. Another marker, VDAC1, is a multifunctional protein that is involved in the regulation of mitochondrial membrane transport. This protein is considered to influence the overall functional state of mitochondria by controlling the flux of metabolites through the outer mitochondrial membrane^36,37^. Immunofluorescent analyses revealed that VDAC1 expression was markedly decreased in PD nigral neurons compared to age-matched controls. In particular, colocalization studies revealed that lower VDAC1 immunoreactivity was found both in the neuronal perikarya with α-synuclein inclusions, as well as within the neuropil displaying swollen α-synuclein aggregates^36^.

Related to this, PINK1 is involved in the regulation of signaling pathways mediating mitochondrial quality control during mitochondrial damage. Indeed, PINK1 is expressed throughout the human brain and it is found in all cell types, with a punctate cytoplasmic immunostaining pattern consistent with mitochondrial localization^38^. Interestingly, the latter study also showed that the immunohistochemical appearance and cellular localization of PINK1 within different brain regions in sporadic PD is indistinguishable from those in the normal human brain^38^. Chronic proteopathic stress in NDDs is also associated with lysosomal dysfunction, which in turn may contribute to the pathological accumulation of misfolded proteins^39^. Impairments in lysosomal-mediated degradation mechanisms, such as reduction in LAMP2 within PD SN^40^, may lead to the accumulation and aggregation of α-synuclein, with deleterious consequences on neuronal homeostasis^41^. These reports on the deficiency of lysosomal markers are also interesting, since the mRNA expression of two markers in the lysosome mediated degradation pathways *LAMP2* and *RUBCN* is paradoxically increased in PD SN (Fig. 3a; GSE7621 and GSE43490).

Lastly, using a multi-pronged approach- including the NCBI GEO datasets highlighted in this article- we have previously reported findings in reflecting neuronal stress response in AD and PD, and potentially are relevant to biomarker discovery. In brief, we showed that in (post-mortem) brains of PD and AD, the expression and activity of eukaryotic elongation-factor 2 kinase (eEF2K) is significantly increased, both at the level of mRNA expression and also at the level of substrate phosphorylation (Immunohistochemistry- IHC detection of p-eEF2, Thr56)^26,27^. eEF2K acts in concert with the energy-sensing cellular machinery, and especially neuronal eEF2K couples local mRNA translation to synaptic activity via phosphorylation of eEF2 (Thr56)^42^. Specifically, the aberrant eEF2K response is reproducible in neuronal cultures under conditions of supra-physiological α-synuclein overexpression (mimicking α-synuclein aggregation), or in a transgenic mouse model of α-synucleinopathy^25^. Another example relevant to biomarker discovery is the regulation of cellular redox homeostasis, since an imbalance between pro-oxidant and anti-oxidant factors is a well known mechanism underlying cellular damage during ageing or in disease states. These processes are modulated by the nuclear factor erythroid 2–related factor 2 (Nrf2), which acts as the master regulator in transcriptional control of pro-survival and anti-oxidant gene expression. In this context, we have reported an aberrant Nrf2-dependent gene expression in PD patient brains, with similar observations in the brains of a transgenic mouse model of α-synucleinopathy. For instance, we found increased mRNA expression of Heme-oxygenase 1, HO-1 (*Hmox1*: an anti-oxidant factor under the transcriptional control of Nrf2) in both PD brains and in the brains of transgenic mice in the presence of widespread α-synuclein aggregation^25^. Both these observations represent an untapped opportunity for novel biomarker discovery in neuronal stress response, as reflected by studies showing higher concentrations of HO-1 in the serum of PD patients^43^.

### The methodological context

Although the availability of omics datasets is seen as advantageous for biomarker discovery, there are inherent features in the biological data which require particular attention, especially in scenarios where multiple sampling of datasets is performed. Moreover, in the context of NDDs, it is also important to consider whether changes in the abundance of given marker(s) are due to alterations in the cellular composition within the brain tissue, or represent adaptive transcriptional response in the regulation of expression^44^. Hence, it is advisable that an analytical plan is in place which includes penalties accounting for multiple sampling, in particular the false discovery rate (FDR) and biological variation across samples^45^.

While no single fit-for-all-purpose statistical approach can be proposed, some guidelines are highlighted below. In the exploratory scenario (e.g., Comparing the Mean value of one marker in one brain region between population A and B), one could start by establishing the null hypothesis (p-value) in pairwise comparison. This could be  followed by assigning the biologically informative variable, which could originate from an independent measure (i.e., multiple datasets, disease relevance and/or experimental validation)^46^. For instance, in our studies on eEF2K cited above, we found that increased gene expression in AD and PD brains was also reflected by substrate phosphorylation (p-eEF2, Thr56)^26,27^. In this scenario, one could also reduce the bias by statistically filtering the data to account for outlier entries and set a threshold criteria in relative abundance (e.g., log(2) fold-change ± 0.25) to assign a presumptive positive status. However, biological processes seldom change in isolation; hence, eventually an approach that involves multiple comparisons will be needed for further validation, and ideally at the biological pathway level. In other words, ‘statistically significant’ differences should also be meaningful in the biological context, i.e., they are also ‘biologically significant’^45^.

The eventual aim should be to minimize the FDR when simultaneously testing minimal hypotheses within an omics dataset, by determining significance thresholds and quantifying the overall error rate, for instance using the Benjamini and Hochberg correction^47^ or variations thereof^48^ (NOTE: When performing global analysis for significant differences within a dataset in the NCBI GEO2R, the users have the option to incorporate Benjamini and Hochberg correction in their analyses- See Fig. S2). This approach allows the investigator to assign an acceptable level (e.g. 5%) of FDR, i.e., any significant finding has 5% chance of being false positive discovery^45^. While a detailed description of these methodologies is beyond the scope of this manuscript, the idea is to perform several pair-wise comparisons, and then ranking the test-derived p-values against different increments of significant thresholds (see examples by Fay, D. S. and Gerow, K.^45^). This would generate a matrix in which it is possible to determine if the p-value for a given comparison is less than the corresponding threshold, hence this is termed a discovery. Through further iterations, the process is continued till a p-value is reached which is higher than the threshold, beyond which all remaining comparisons are considered not significant. Several refinements to this approach have also been proposed, such as p-value weighting^49^, stratified FDR^50,51^ and functional FDR with informative variable^46^. Lastly, there is no fixed rule that in a given dataset, all comparisons should be performed. Instead, it is acceptable that a subset of comparisons is decided *a priori* to analyses, which are either biologically interesting and/or relevant to the main focus of a study^45^.

### Additional resources

#### Microarray datasets

To facilitate cross-comparison analyses across NDDs, we have compiled a list of studies on the brain expression profiles in the Table S3: Excel file in the figshare repository (refer to the section: Data Availability), covering AD, PD, HD, MS and ALS^14,17,52–72^. In addition to targeted queries, it would be highly informative to apply high-content machine learning methodologies towards uncovering common mechanisms in NDDs, as shown by the studies on GEO microarray datasets from blood samples (blood transcriptome)^8^. This elegant report revealed that perturbations in several cellular pathways (e.g., mitochondrial function, immune response, protein synthesis) are a shared feature in common NDDs^8^. Another transcriptome resource worthwhile to mention is the NeuroTransDB, which contains curated metadata obtained from studies in AD patients, as well as cellular and animal studies from published literature in AD^9^. The potential utility of the latter resource lies in the fact that alterations in the transcriptome consequent to a targeted manipulation in cellular and animal studies (e.g., genetic deletion, overexpression etc.) can be cross-referenced to patient-derived datasets.

#### CSF proteome

In this section, we briefly highlight additional omics resources that can be useful in translating gene expression profiling into protein expression and/or secretion. We demonstrate this by presenting the findings on the detection of some of the select markers (Table S2) in the CSF-based studies in NDDs. For instance, elevated levels of LAMP2 levels have been detected (compared to control subjects) by western immunoblotting analyses of CSF, both in AD^73^ and in PD^74^. A highly valuable, and easy to use, online portal to access the CSF proteomics is the CSF proteome Resource developed by researchers at the University of Bergen, Norway (refs. ^12,13^ also see weblink to the portal in Table 1). The portal contains 133 published datasets derived from CSF-based proteomics studies including PD, AD, ALS and MS. To illustrate an example, when data for the GSS abundance in CSF are queried (Uniprot: P48637), the portal shows that higher levels of GSS are detected in AD (with corresponding reference to the reporting study^75^), while no significant differences are found in a subset of MS cases (with reference to the study^76^). Query for LAMP2 (Uniprot: P13473) show no significant overall changes in PD, AD and MS, except one study showing higher detection in MS (Table S4: Excel file in the figshare repository). Furthermore, targeted queries on the CSF portal revealed alterations in the levels of SNCA, MAPT, UCHL1, PARK7 (DJ-1) NEFL, GSS, GGCT, GCLC, GGT1, SOD1, ATP1A1, ATP2A2, RYR1, GRM5 and LAMP2, as reported by one or multiple studies^75–91^. These findings are summarized in the Table S4 and uploaded to the figshare repository (refer to the section: Data Availability). In addition to the datasets available through the CSF portal, several ultra deep proteome studies have been published recently^75,92–95^, and the associated datasets are accessible through the AD Knowledge portal (Table 1). Combining advanced methods in mass spectrometry and systems biology approach, these studies are among the most extensive resources published to-date, including phosphoproteomics in AD^92^. Overall, the data provide further evidence regarding defective energy metabolism in response to the proteopathic stress in neurodegeneration^93,95^, with novel insights regarding potential markers of AD progression^75,92^ and common mechanisms in AD and PD^94^.

#### Recent advances in web-based platforms

There has been significant activity in the field that has enhanced the capabilities of research community to access larger datasets and perform cross-comparison studies, with metadata accessible through online platforms. For instance, the Parkinson’s Progression Markers Initiative (PPMI) sponsored by the Michael J. Fox Foundation is one of the largest longitudinal, observational, and multi-center venture providing open-access data on the progression of clinical features, imaging outcomes, and biologic and genetic markers across all stages of PD (including CSF markers, weblink in Table 1). Very recently, the European Platform for Neurodegenerative Diseases (EPND), a large consortium supported by the European Unions’ Innovative Medicine Initiative, has also released its catalogue with metadata on 60 cohorts across Europe. Another useful resource to consider is Agora, an open-access portal funded by the National Institute on Aging (Table 1). On this portal, the users can access transcriptomic, proteomic, and metabolomic evidence to assess genetic association of candidate markers with AD, including the correlation of mRNA abundance within different brain regions to relative protein expression. Also, the outcomes of an elegant study involving spatial transcriptomics analyses in spinal cord sections from ALS patients and a mouse model are also available online as an open-access resource (see Table 1 for the weblink: ALS-ST)^96^.

#### A note on the sources for data analyses and visualization

Lastly, for the readers with little/no bioinformatics knowledge, there are open-access sources for performing data analyses (e.g., GSEA GenePattern, see Table 1), as well as for detailed annotations and pathway enrichment (e.g., Metascape developed by^97^, Table 1). To illustrate this point, we probed the gene ontology and pathway enrichment for the gene families listed in Table S2 using Metascape. As expected, there is preponderance of pathways associated with NDDs (Fig. S8), visualized in the form of charts showing gene clustering and protein-protein interaction, which can further be explored with the online interactive cytoscape feature. The users can download all the charts and heatmaps (e.g., for publication), along with additional features of the data such as disease association of the candidate genes and information on transcription factors etc.

In conclusion, with increasing standardization of the data collection methodologies and refined algorithms for integrating clinical outcomes with measurements of candidate biomarkers (e.g., in biological fluids and/or brain imaging), the availability of novel bioassays for NDDs is a realizable outcome in near future. This will significantly boost efforts not only for diagnosis, enrollment, stratification and monitoring of patients in clinical studies, but also strengthen ventures aimed at mechanism-based drug discovery. Moreover, when taken in conjunction with the existing markers of pathology^7^, such insights may also help settle crucial debates on the pathogenic significance of protein aggregation in the nervous system during the progression of NDDs. We hope that this Resource article will boost these efforts, and importantly facilitate the education and training of younger researcher to fully realize the potential of the listed resources.

## Supplementary information

SDATA-23-00266A_Supplementary Information

## Data Availability

The transcriptomics datasets analyzed during this study can be accessed on the NCBI GEO portal (https://www.ncbi.nlm.nih.gov/geo/geo2r), using the accession provided in the referenced DOI^98–124^. Otherwise, all the data analyzed during this study are included in the main manuscript or the associated Supplementary Information files (online PDF). Additional supplementary files have been uploaded on the figshare repository, and include the following: (i) DATA BEHIND FIGURES^125^ (i.e., Data on individual gene markers presented in the Figs. 1–3 and S4–S7), (ii) ADDITIONAL EXCEL FILES^126^, containing Table S3 (additional microarray datasets with GEO accession), Table S4 (CSF proteome profiling covering select markers), SUPPLEMENTARY EXCEL FILE 1, containing unique probe IDs for the platform GPL570 (for Dataset GSE7621 and Dataset GSE20146), GPL6104 (for Dataset GSE26927) and GPL6480 (for Dataset GSE43490) and SUPPLEMENTARY EXCEL FILE 2, containing global expression profile of the top altered genes (differential expression between control and PD samples) in the PD datasets GSE7621, GSE43490 and GSE20146.

## Code availability

No custom code was used or generated, otherwise the details on the open-access platforms used are listed in Table 1.
