# Supplementary material for: A beginner’s guide into curated analyses of open access datasets for biomarker discovery in neurodegeneration
Source: Sci Data. 2023 Jul 6;10:432. doi: 10.1038/s41597-023-02338-1 (PMC10325954; doi:10.1038/s41597-023-02338-1)
Supplement: Supplementary file 1 — SDATA-23-00266A_Supplementary Information [file 41597_2023_2338_MOESM1_ESM.pdf]

SUPPLEMENTARY INFORMATION

TITLE: A beginner's guide into curated analyses of open access datasets for biomarker discovery in neurodegeneration

Authors: Diana Gomes Moreira[1], Asad Jan[2¶].

Author Affiliations:

[1] Department of Clinical Medicine, Palle Juul-Jensens Boulevard 165, DK-8200 Aarhus N, Denmark

[2] Department of Biomedicine, Aarhus University, Høegh-Guldbergs Gade 10, DK-8000, Aarhus C, Denmark

[§]**Address correspondence to**:

Asad Jan, Department of Biomedicine, Høegh-Guldbergs Gade 10, 8000 Aarhus, DK-8000 Aarhus C, Denmark. Tel. +45 87 15 36 92; Email: ajan@aias.au.dk; ORCID ID: 0000-0002-3636-0070

SUPPLEMENTARY MATERIAL IN THIS FILE (excluding large excel files):

- FIGURES S1-S8
- TABLE S1-S2

FIGURE S1

a) PICTORAL OVERVIEW OF CURATED ANALYSES USING GEO2R

1) Search the datasets on https://www.ncbi.nlm.nih.gov/geo/, by entering the dataset ID (e.g., GSE7621)

2) On the next page, scroll to the bottom and click: "Analyze with GEO2R", it will load the GEO2R interface

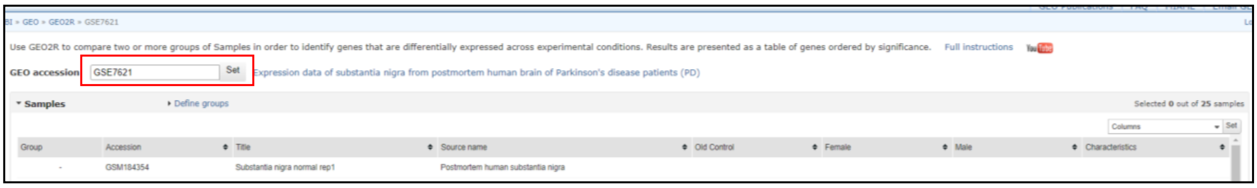

3) Then, Click on "Profile Graph"

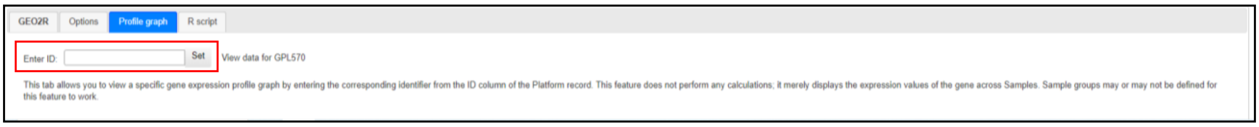

4) In the box "Enter ID", enter the unique probe ID for a marker of interest and click "Set". It will load a plot as shown below. Next to the plot, click "Sample values"

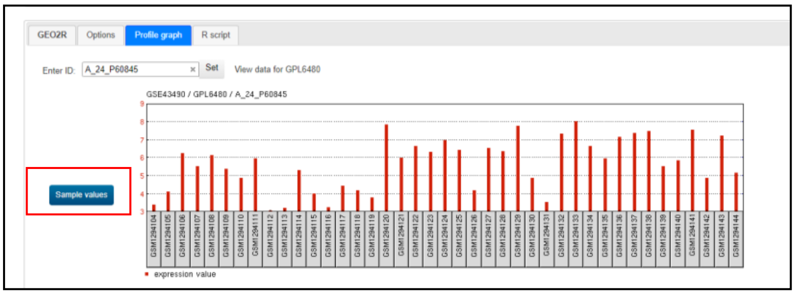

5) A pop-up table (highlighted by blue box below) will be displayed

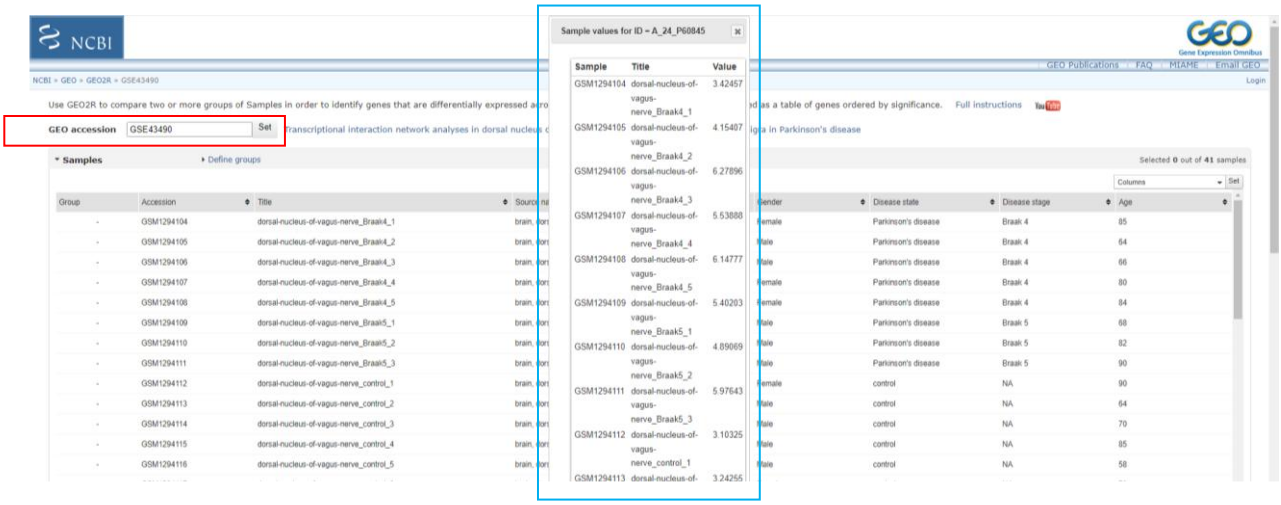

6) Copy the table and transfer as text into a data processing software.

NOTE: A quicker way to navigate all datasets in the same window is the following: Scroll to the top of the page and enter dataset accession (e.g. GSE20146, as shown in the red box above), and click "Set". This action will load the new dataset. Then, enter the unique probe ID for the gene(s) of interest under "Profile Graph" and repeat the process shown in steps 3-6 above.

FIGURE S2

a) PICTORAL OVERVIEW OF GLOBAL ANALYSES USING GEO2R

1) Search the datasets on https://www.ncbi.nlm.nih.gov/geo/, by entering the dataset ID (e.g., GSE7621)

2) On the next page, scroll to the bottom and click: "Analyze with GEO2R", it will load the GEO2R interface

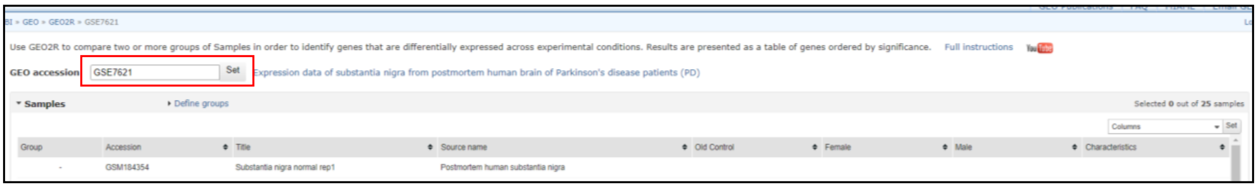

3) Then, click on "Define Groups" and assign each sample to the respective group. This is done by selecting the corresponding samples (while holding SHIFT key) and selecting the group.

In the example below, we have created a Control and PD group in the GSE7621.

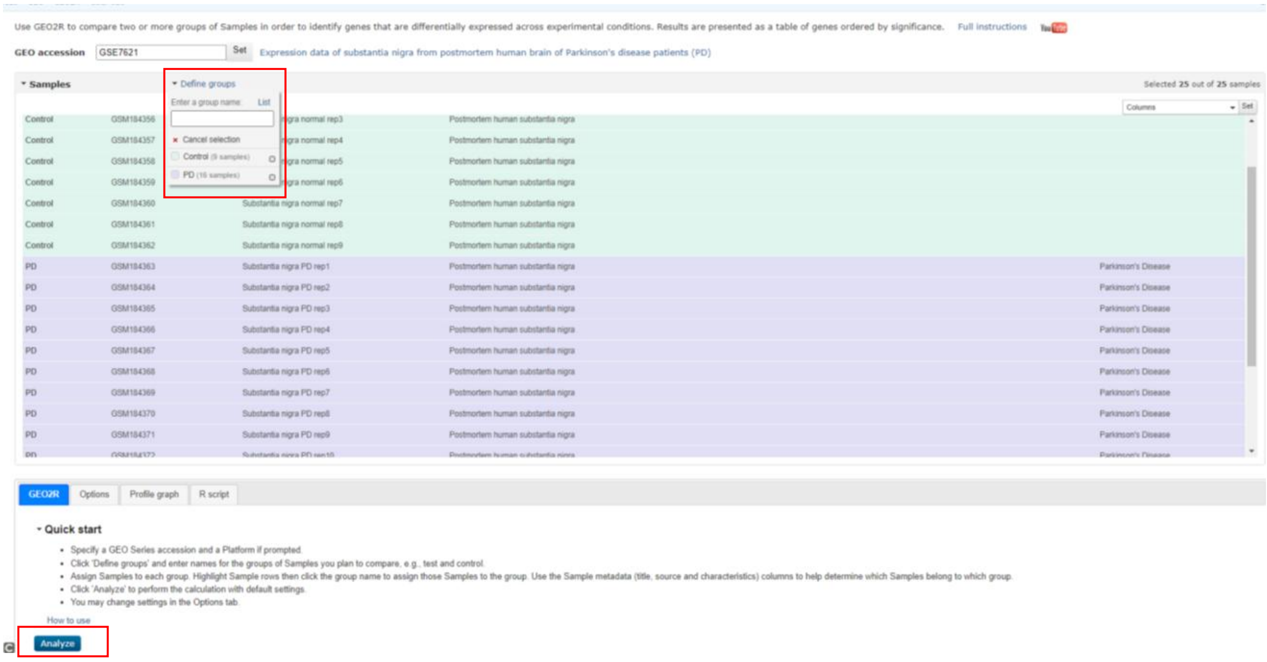

4) Once the groups are assigned, click "Analyze", (highlighted in STEP 3 above). It will generate a table with differential gene expression profiles and plots for visualization (all of which can be downloaded)

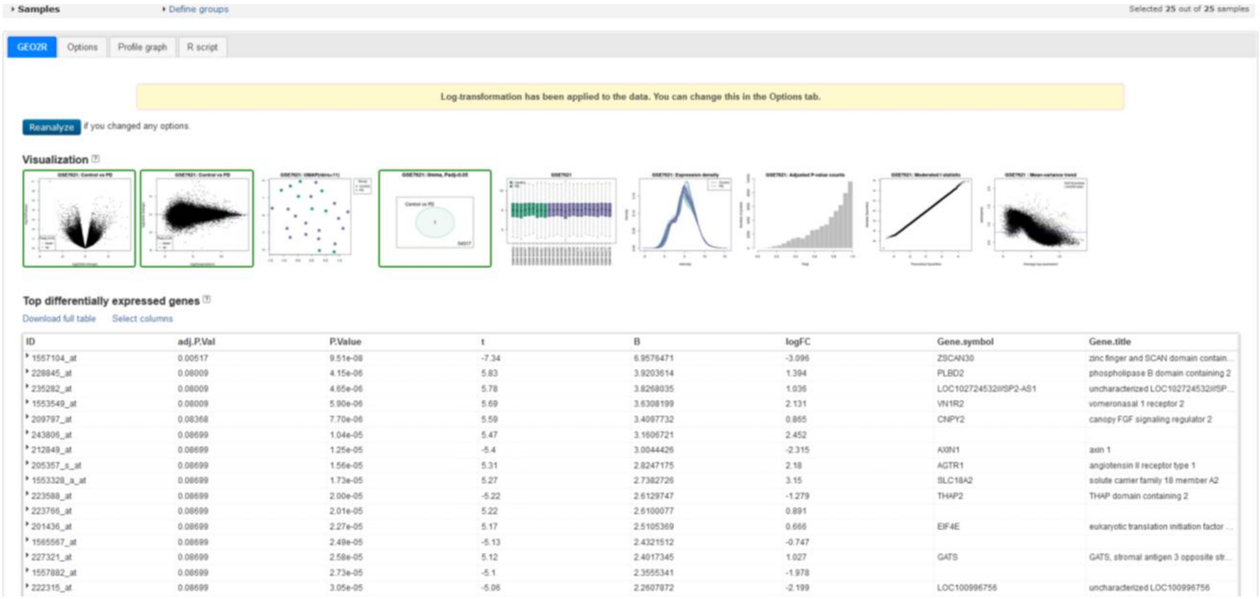

5) The user can also modify the settings for statistical analyses and data plots, the defaults are shown below

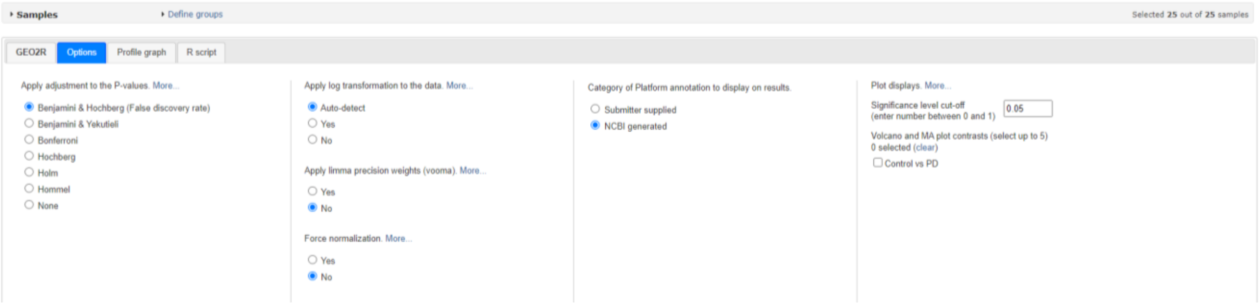

FIGURE S3

a) PICTORAL OVERVIEW OF CURATED ANALYSES OF CSF PROTEOMICS

1) Access the weblink https://proteomics.uib.no/csf-pr/ and click "Search protein data"

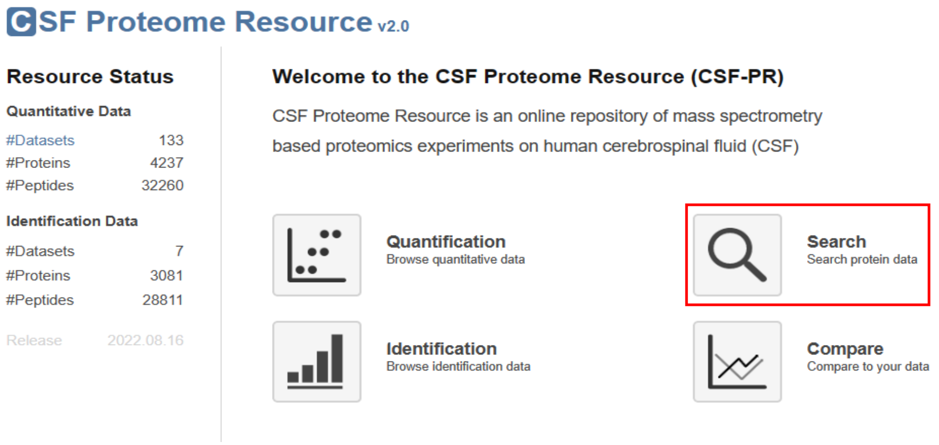

2) In the Search box, enter the unique identifier (e.g., Uniprot ID), select the input type (e.g., "Protein Accession") and the disease category, and then click "Search".

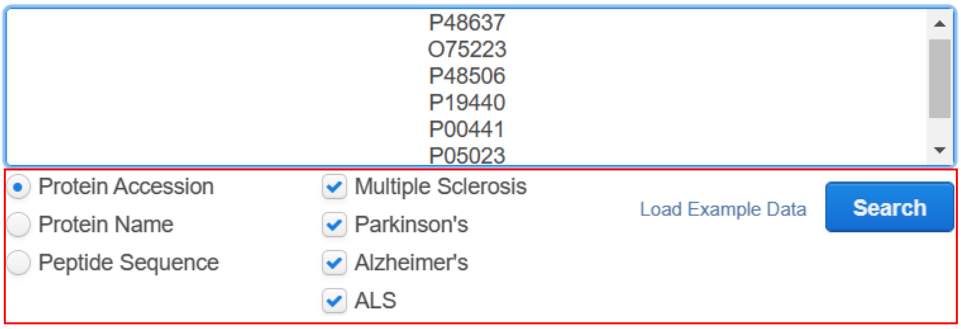

3) A graphical overview of the results will be displayed, which can be viewed all together or for an individual marker. In the chart below, out of the 7 marker queries, 6 were found within the database and 1 was not found.

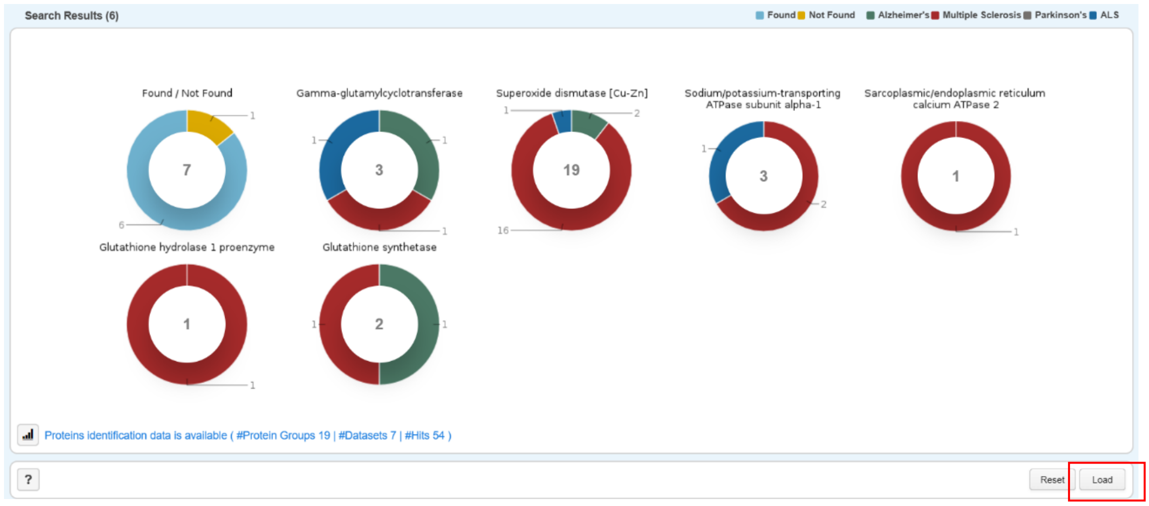

4) In the chart shown under Step 3, the number in the center for each marker (circle) indicates the number of individual datasets a given marker has been reported. The relevant datasets are also color coded with reference to the disease category. To view the linked data, click Load. A new interactive interface will appear, as shown below.

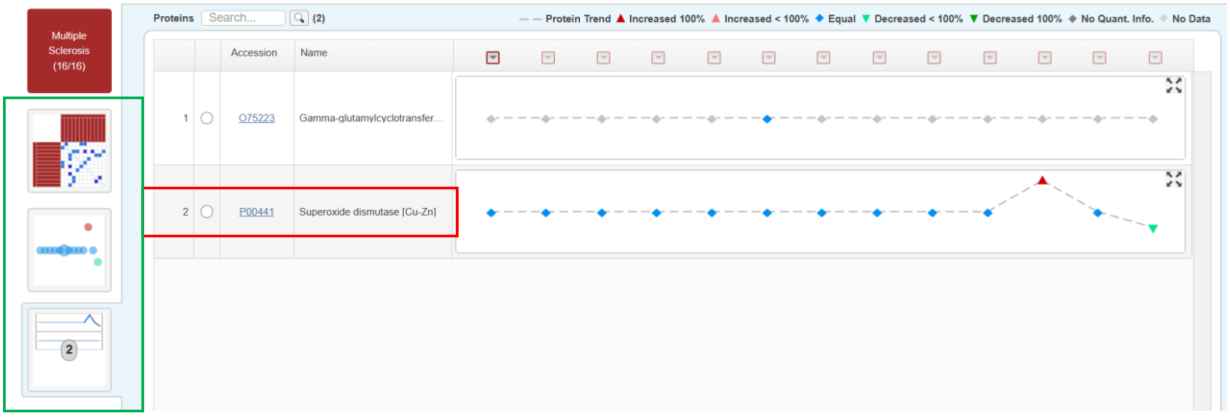

In this interface, the icons on the left side (green box above) are arranged as 3 panels.

A- Disease comparison, datasets arranged across the disease categories (and sub-categories). Here datasets can be included or excluded based on the intended patient populations.

B- Protein Overview, pictoral representation of the protein detection being increased (red circle), decreased (green circles) or Equal/unaltered (blue circles). This menu is further option of filtering, ie. de-selecting markers whose levels were reported to be equal/unaltered between control and disease conditions

C- Protein Table, based on the selection and filtering in Disease comparison and Protein overview, this option is linked to specific studies and reported trend

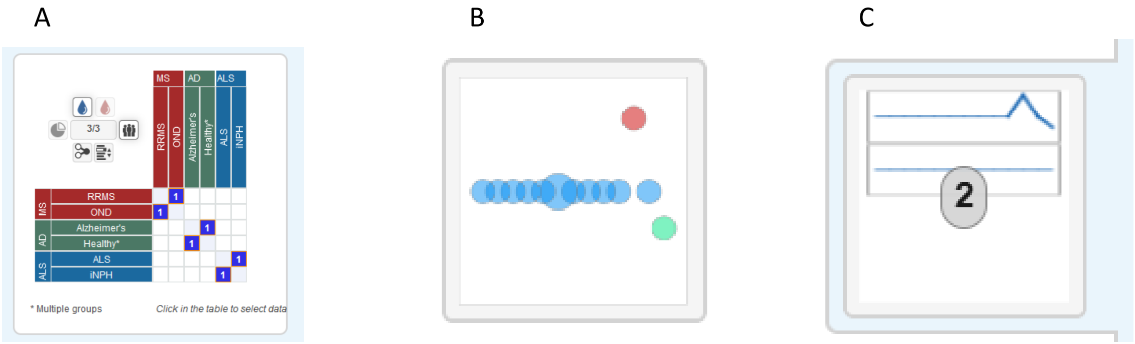

NOTE: Panel A also contains an overview of the datasets and linked publications

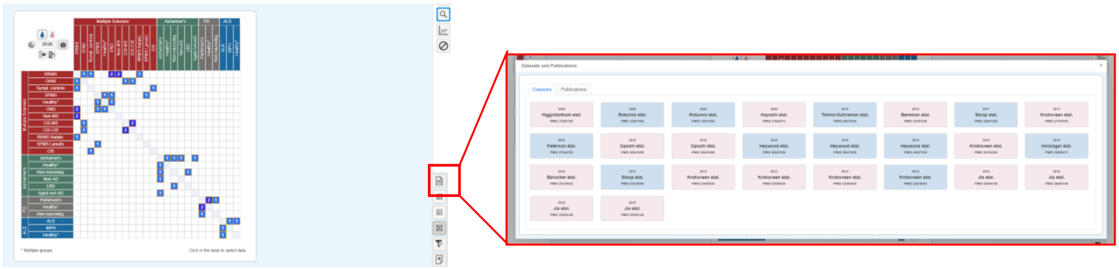

5) In the Protein Table, select the desired marker. A new sub-Menu “Protein Details” will appear (highlighted in the red box below).

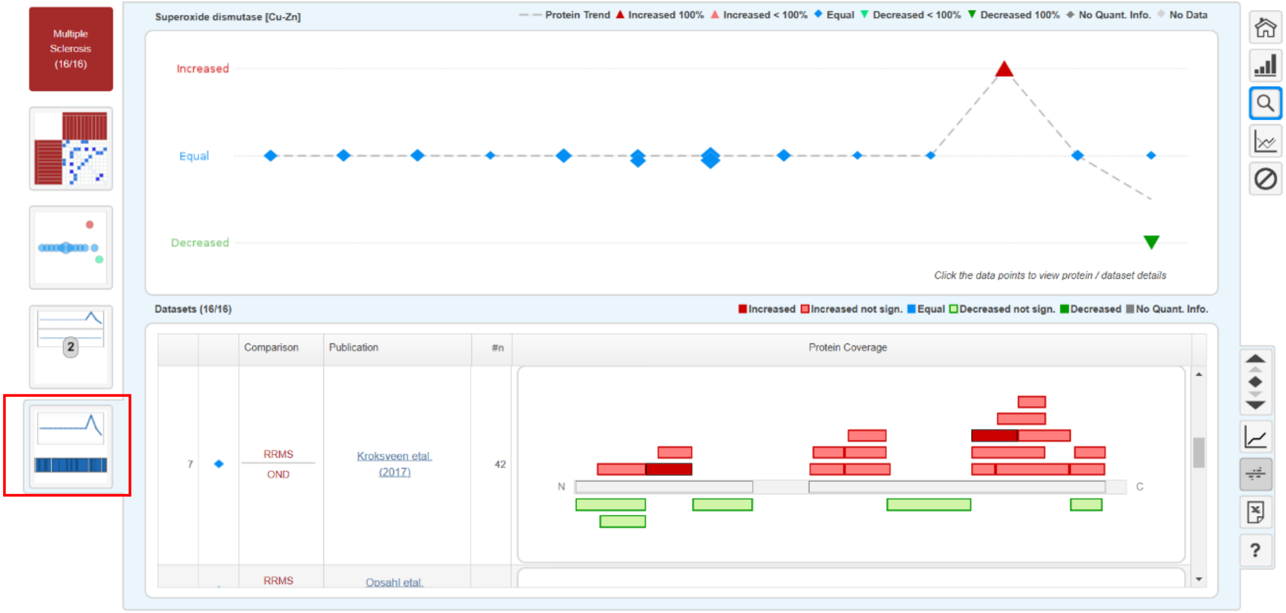

6) Clicking “Protein Details” will display the linked dataset(s), including peptide information

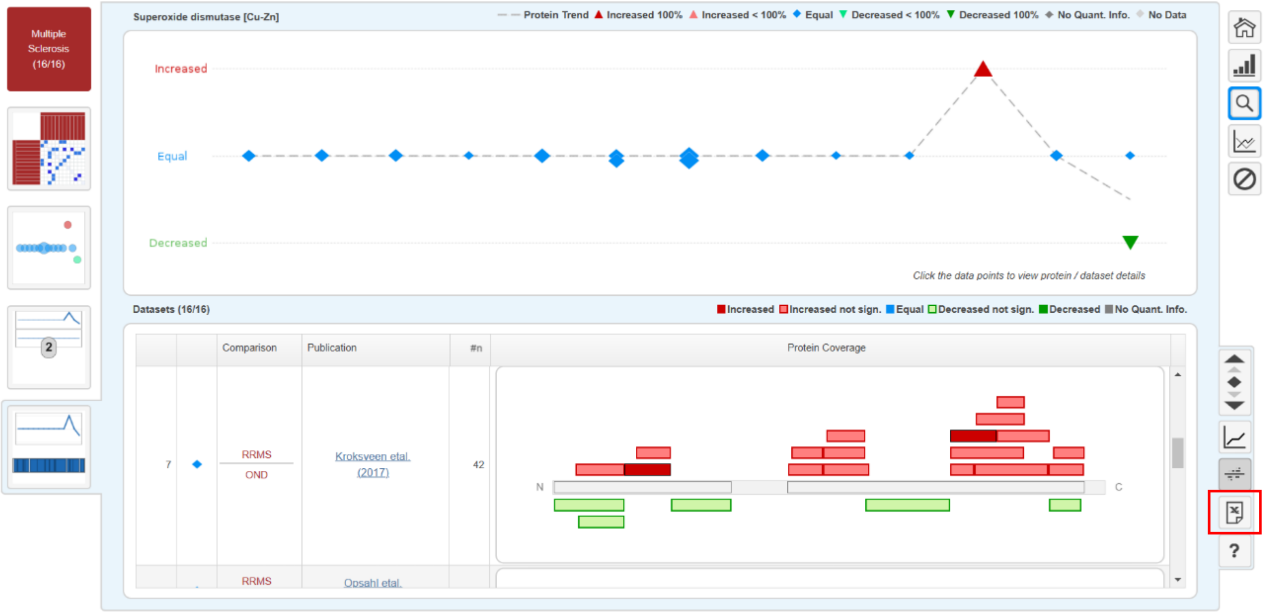

7) Selected data can be downloaded as an Excel file, by clicking the icon highlighted in the red.

Fig. S4

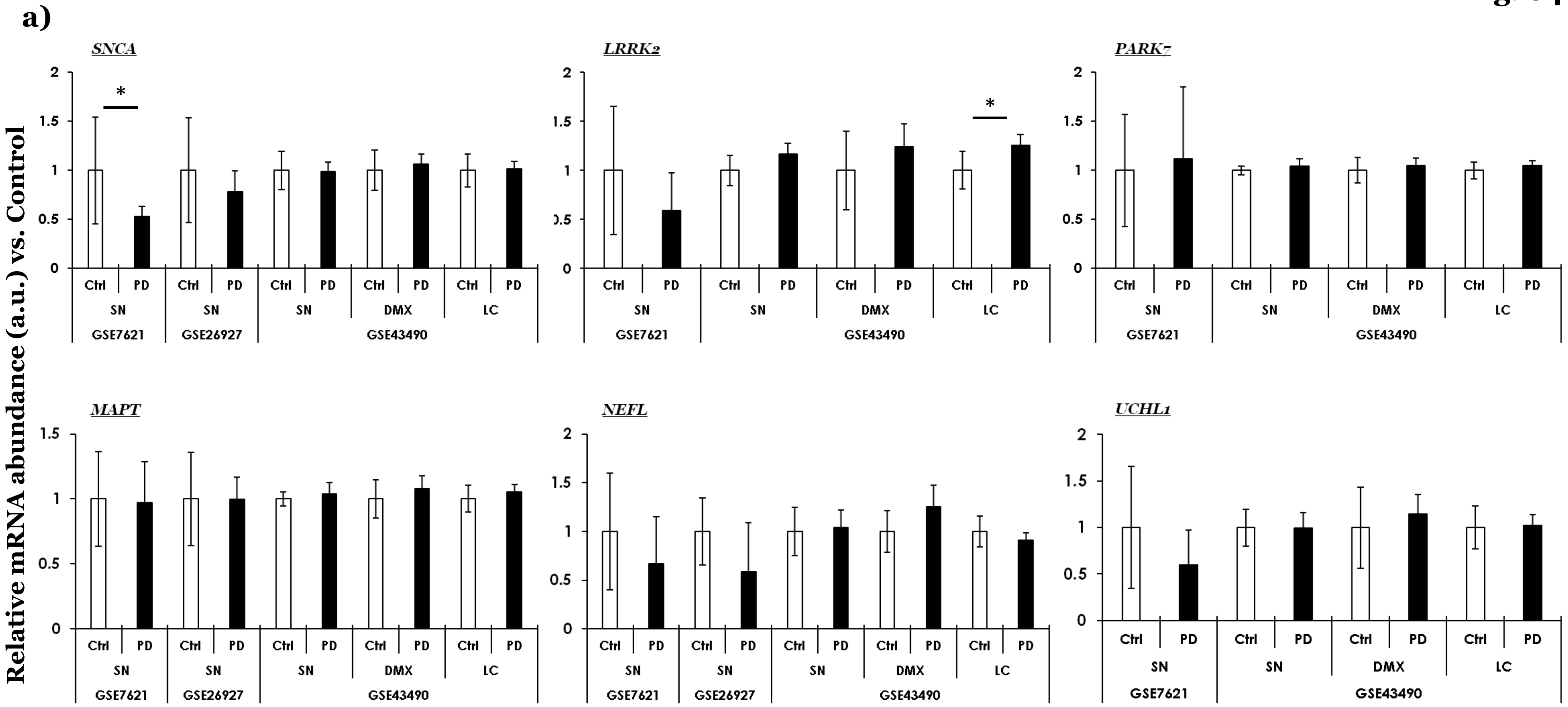

Figure S4. Curated gene expression analyses of selected factors associated with neurodegeneration within GEO microarray datasets in PD. **(a)** Alpha-synuclein (gene symbol, *SNCA*), Leucine-rich repeat serine/threonine-protein kinase 2 (gene symbol, *LRRK2),* Parkinson disease protein 7 (DJ-1) (gene symbol, *PARK7),* Microtubule-associated protein tau (gene symbol, *MAPT),* Neurofilament light polypeptide (gene symbol, *NEFL)* and Ubiquitin carboxyl-terminal hydrolase isozyme L1 (gene symbol, *UCHL1)***.** The values across the datasets are expressed relative to the controls in each microarray dataset, i.e., mean value of control samples=1 (a.u., arbitrary units). Error bars represent standard deviation of the mean, s.d. Pair-wise comparisons were assessed by Mann-Whitney test- only significant differences (*=p≤0.05, **=p≤0.01, ***=p≤0.005) are highlighted. The number of controls and cases, microarray platforms are included in Table S1. Unique probe IDs within each dataset are included in Table S2. ***Legend***: Ctrl (controls); PD (Parkinson disease); SN (*substantia nigra*); DMX (*dorsal motor nucleus of vagus*), LC (*locus coeruleus)*; GPi (*globus pallidus interna*).

Fig. S5

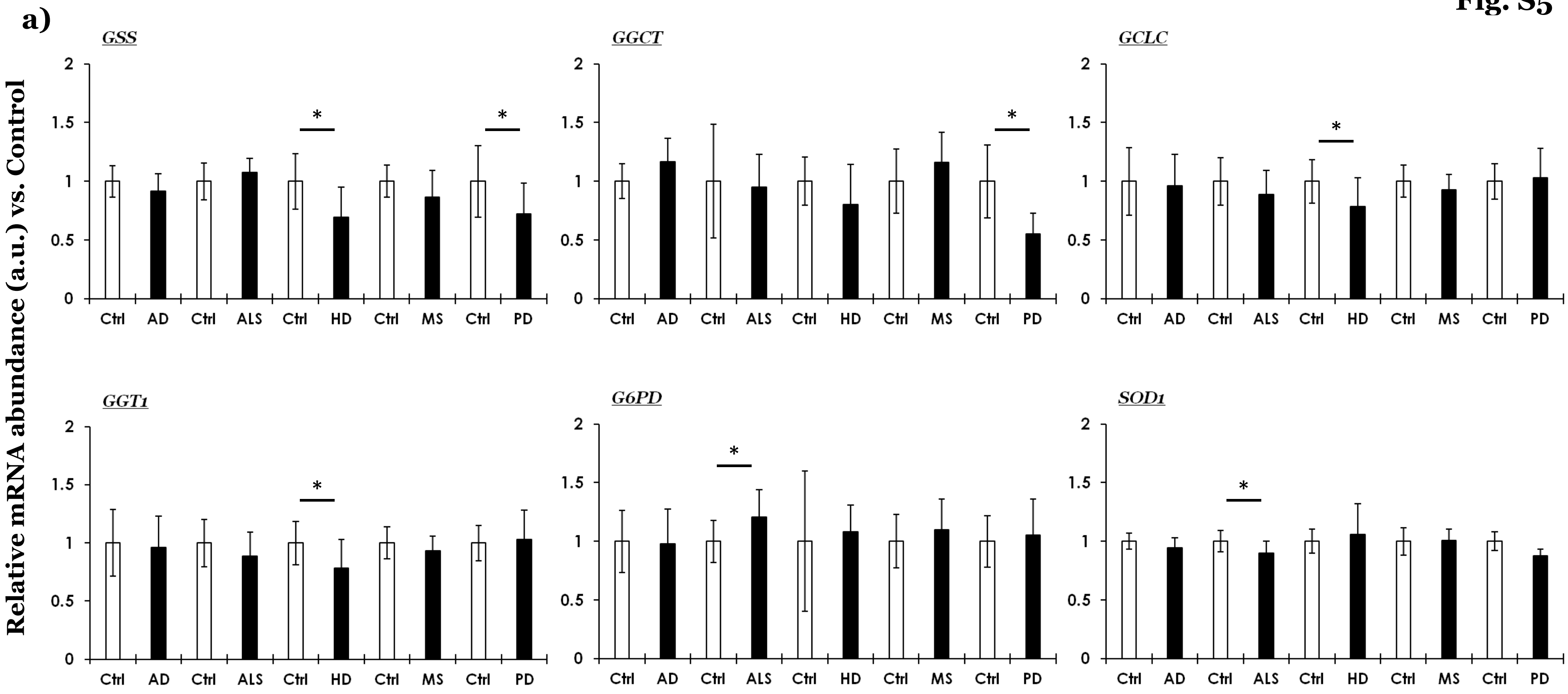

Figure S5. Curated gene expression analyses of glutathione biogenesis related factors within GEO microarray dataset GSE26927 across common neurodegenerative diseases (and Multiple sclerosis, a demyelinating disease). (a) Glutathione synthetase (gene symbol, *GSS*), gamma-glutamylcyclotransferase (gene symbol, *GGCT),* gamma-glutamylcysteine synthetase (gene symbol, *GCLC),* gamma-glutamyltransferase/transpeptidase 1 (gene symbol, *GGT1),* glucose-6-phosphate dehydrogenase (gene symbol, *G6PD)* and superoxide dismutase (gene symbol *SOD1)***.**

The values across the dataset are expressed relative to the controls in each disease condition, i.e., mean value of control samples=1 (a.u., arbitrary units). Error bars represent standard deviation of the mean, s.d. Pair-wise comparisons were assessed by Mann-Whitney test- only significant differences (*=p≤0.05, **=p≤0.01, ***=p≤0.005) are highlighted. The number of controls and cases, microarray platforms and original studies are included in Table S1. Unique probe IDs within each dataset are included in Table S2.

Legend: Ctrl (controls); Alzheimer disease (AD); Motor neurone disease (ALS); Huntington disease (HD), and Multiple sclerosis (MS).

Fig. S6

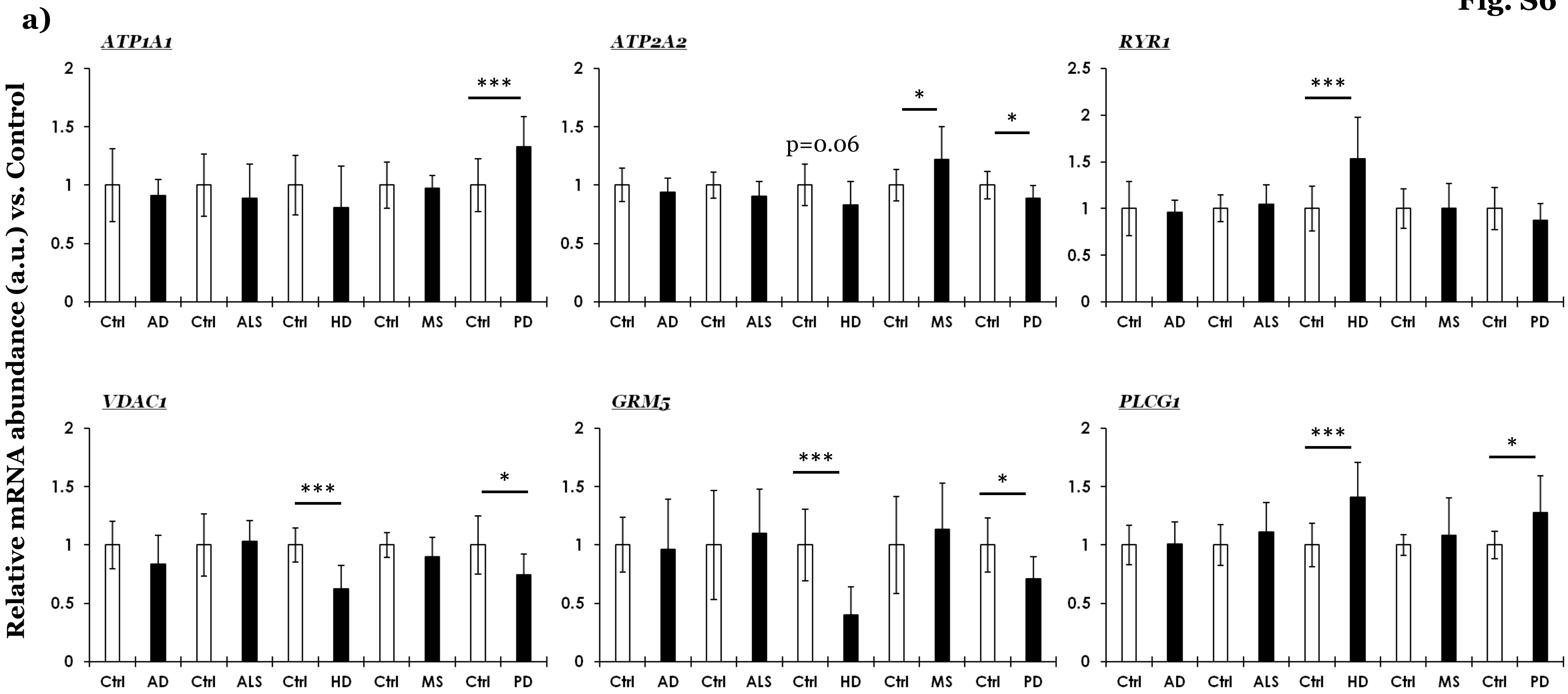

Figure S6. Curated gene expression analyses of neuronal excitability/calcium signaling related factors within GEO microarray dataset GSE26927 across common neurodegenerative diseases (and Multiple sclerosis, a demyelinating disease). (a) Sodium-potassium ATPase, catalytic subunit alpha-1 (*ATP1A1*), sarcoplasmic/endoplasmic reticulum (ER) Ca^{2+} transporting ATPase 2, alias SERCA2 (*ATP2A2*), ryanodine receptor 1 (*RYR1*), voltage dependent anion channel 1 (*VDAC1*), glutamate metabotropic receptor 5 (*GRM5*), and phospholipase c gamma 1 (*PLGC1*).

The values across the dataset are expressed relative to the controls in each disease condition, i.e., mean value of control samples=1 (a.u., arbitrary units). Error bars represent standard deviation of the mean, s.d. Pair-wise comparisons were assessed by Mann-Whitney test- only significant differences (*=p≤0.05, **=p≤0.01, ***=p≤0.005) are highlighted. The number of controls and cases, microarray platforms and original studies are included in Table S1. Unique probe IDs within each dataset are included in Table S2.

Legend: Ctrl (controls); Alzheimer disease (AD); Motor neurone disease (ALS); Huntington disease (HD), and Multiple sclerosis (MS).

Fig. S7

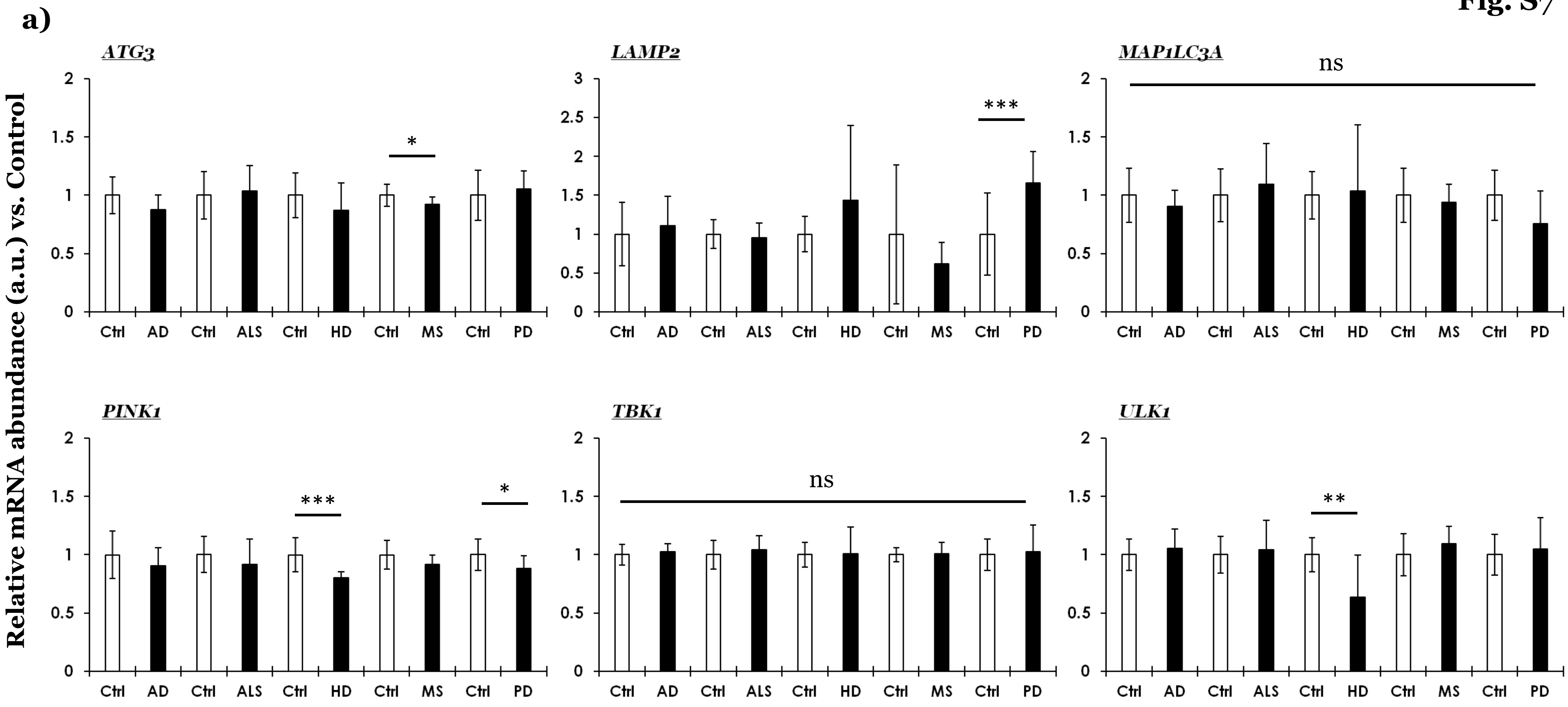

Figure S7. Curated gene expression analyses of factors involved in the regulation of autophagy within GEO microarray dataset GSE26927 across common neurodegenerative diseases (and Multiple sclerosis, a demyelinating disease). (a) Autophagy Related 3 (*ATG3)*, lysosomal associated membrane protein 2 (*LAMP2)*, microtubule associated protein 1 Light Chain 3, alias LC3 alpha (*MAP1LC3*,) PTEN Induced Kinase 1 (*PINK1)*, Unc-51 like autophagy activating kinase 1 (*ULK1*) and TANK binding kinase 1 (*TBK1).* The values across the dataset are expressed relative to the controls in each disease condition, i.e., mean value of control samples=1 (a.u., arbitrary units). Error bars represent standard deviation of the mean, s.d. Pair-wise comparisons were assessed by Mann-Whitney test- only significant differences (*=p≤0.05, **=p≤0.01, ***=p≤0.005) are highlighted. The number of controls and cases, microarray platforms and original studies are included in Table S1. Unique probe IDs within each dataset are included in Table S2. ***Legend:*** Ctrl (controls); Alzheimer disease (AD); Motor neurone disease (ALS); Huntington disease (HD), and Multiple sclerosis (MS).

Fig. S8

a)

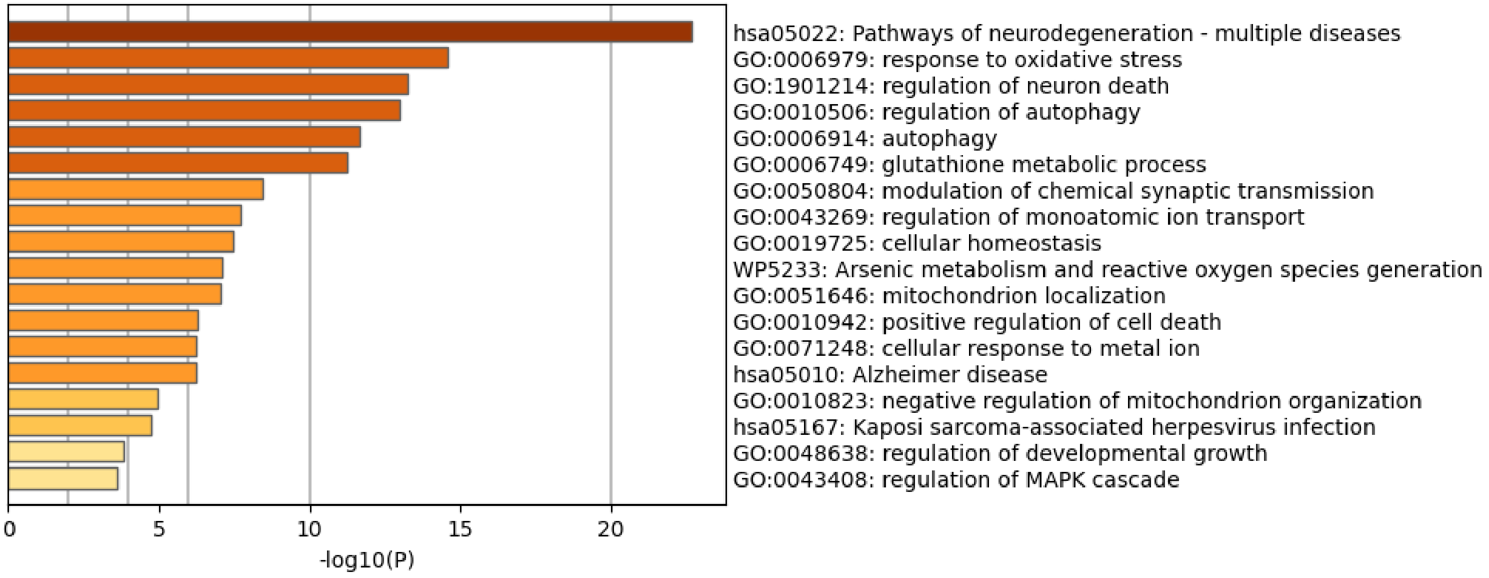

b)

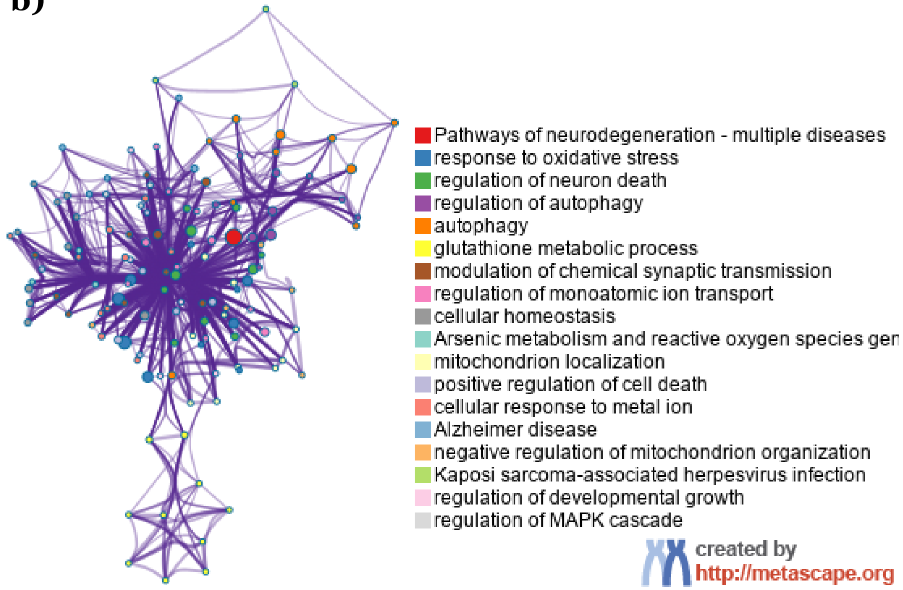

c)

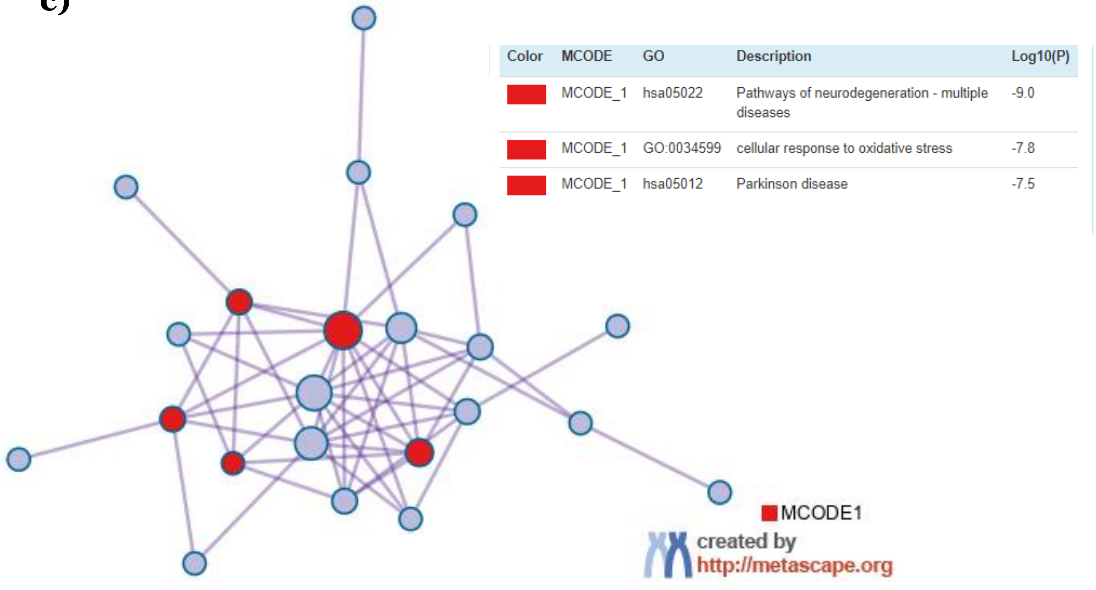

Figure S8. Pathway enrichment and protein-protein interactions (of genes in Table 2) visualized using Metascape7.

(a) Bar graph of enriched terms across input gene lists, colored by p-values. **(b)** Network of enriched terms, colored by cluster.

(c) Protein-protein interaction network and MCODE components identified in the gene lists.

Table S1. Details of the studies used in Figures 1-3 and S4-S7

**GEO accession**	**GSE7621**	**GSE43490**	**GSE20146**	**GSE26927**
**Samples**	SN: Ctrl (n=9), PD (n=16)	SN: Ctrl (n=6), PD (n=8)<br>DMX: Ctrl (n=5), PD (n=8)<br>LC: Ctrl (n=7), PD (n=8)	GPi : Ctrl (n=10), PD (n=10)	SN : Ctrl (n=7), PD (n=12)<br>Ent. Ctx: Ctrl (n=6), AD (n=12)<br>Cerv. SC: Ctrl (n=7), ALS (n=9)<br>Caudate: Ctrl (n=10), HD (n=10)<br>Front. gyri: Ctrl (n=10), MS (n=10)<br>Temp. lobe: Ctrl (n=10), SZ (n=10)
**Platform**	GPL570 Affymetrix Human Genome U133	GPL6480 Agilent-014850 Whole Human Genome	GPL570 Affymetrix Human Genome U133 Plus	GPL6255 Illumina humanRef-8 v2.0 expression beadchip
**Reference in the text**	*Lesnick, T.G. et al.,*<br>*Ref, 14*	*Corradini, B.R. et al.,*<br>*Ref, 15*	*Zheng, B. et al.,*<br>*Ref, 16*	*Durrenberger, P.F. et al.,*<br>*Ref, 17*

Abbreviations:

GSE7621- Ctrl (control), PD (Parkinson Disease), SN (*substantia nigra*).

GSE43490- Ctrl (control), PD (Parkinson Disease), SN (*substantia nigra*), DMX (*dorsal motor nucleus CN. X*), LC (*locus coeruleus*).

GSE20146- Ctrl (control), PD (Parkinson Disease), GPi (*globus pallidus, interna*).

GSE26927- Ctrl (control), PD (Parkinson Disease), AD (Alzheimer Disease), ALS (Amyotrophic Lateral Sclerosis), HD (Huntington Disease), MS (Multiple Sclerosis), SZ (Schizophrenia), SN (*substantia nigra*), Ent. Ctx (*entorhinal cortex*), Cerv. SC (*cervical spinal cord*), Front. Gyri (grey matter lesions in *frontal gyri*), Temp. lobe (*temporal lobe*, left area BA22).

Table S2. Selected markers, Unique probe IDs and Putative function

Gene symbol	Uniprot	Protein	Unique Probe ID		
			GPL570	GPL6104	GPL6480
SNCA	P37840	Alpha-synuclein	204467_s_at	ILMN_2235	A_23_P29939
PARK7	Q99497	Parkinson disease protein 7 (DJ-1)	200006_at	NOT FOUND	A_23_P74740
LRRK2	Q5S007	Leucine-rich repeat serine/threonine-protein kinase 2	201387_s_at	NOT FOUND	A_23_P132956
UCHL1	P09936	Ubiquitin carboxyl-terminal hydrolase isozyme L1	201387_s_at	NOT FOUND	A_23_P132956
MAPT	P10636	Microtubule-associated protein tau	203930_s_at	ILMN_137552	A_24_P224488
NEFL	P07196	Neurofilament light polypeptide	221801_x_at	ILMN_22054	A_23_P71492
GSS	P48637	Glutathione Synthetase	201415_at	ILMN_23541	A_23_P210920
GGCT	O75223	Gamma-Glutamylcyclotransferase	215380_s_at	ILMN_2391	A_23_P42695
GCLC	P48506	Glutamate-Cysteine Ligase Catalytic Subunit	1555330_at	ILMN_10857	A_23_P145114
GGT1	P19440	Gamma-Glutamyltransferase 1	208284_x_at	NOT FOUND	A_23_P154986
G6PD	P11413	Glucose-6-Phosphate Dehydrogenase	202275_at	ILMN_1761	A_23_P34093
SOD1	P00441	Superoxide Dismutase 1	200642_at	ILMN_14302	A_23_P154840
ATP1A1	P05023	ATPase Na+/K+ Transporting Subunit Alpha 1	220948_s_at	ILMN_23777	A_23_P1072
ATP2A2	P16615	SERCA2	239996_x_at	ILMN_19965	A_23_P53603
RYR1	P21817	Ryanodine Receptor 1	205485_at	ILMN_11895	A_23_P78867
VDAC1	P21796	Voltage Dependent Anion Channel 1	212038_s_at	ILMN_12620	A_32_P163169
GRM5	P41594	Glutamate Metabotropic Receptor 5	214217_at	ILMN_20911	A_24_P83899
PLCG1	P19174	Phospholipase C Gamma 1	216551_x_at	ILMN_3773	A_23_P254801
ATG3	Q9NT62	Autophagy Related 3	220237_at	ILMN_18856	A_23_P212706
LAMP2	P13473	Lysosomal Associated Membrane Protein 2	203041_s_at	ILMN_26730	A_23_P217447

MAP1LC3A	Q9H492	Microtubule Associated Protein 1 Light Chain 3 Alpha (LC3)	224378_x_at	ILMN_3421	A_23_P154786
PINK1	Q9BXM7	PTEN Induced Kinase 1	209018_s_at	ILMN_27027	A_23_P23194
RUBCN	Q92622	Rubicon Autophagy Regulator	212733_at	NOT FOUND	A_23_P304171
ULK1	O75385	Unc-51 Like Autophagy Activating Kinase 1	209333_at	ILMN_2158	A_23_P72550
TBK1	Q9UHD2	TANK Binding Kinase 1	218520_at	ILMN_11417	A_23_P44768

Table 2 continued on the next page...

(Table 2 continued) Summary of function:

Alpha-synuclein: Neuronal protein putatively involved in synaptic activity, whose misfolding and aggregation is associated with neurodegeneration.

Parkinson disease protein 7 (DJ-1): A multifunctional protein that protects cells against oxidative stress and cell death, acting as redox-sensitive chaperone

Leucine-rich repeat serine/threonine-protein kinase 2: A kinase that phosphorylates a diverse range of proteins involved in synaptic plasticity and autophagic flux

Ubiquitin carboxyl-terminal hydrolase isozyme L1: A cellular mediator of involved in intracellular processing of ubiquitin precursors and of ubiquitinated proteins.

Microtubule-associated protein tau: A crucial cytoskeletal protein that promotes microtubule assembly, stability and neuronal polarity

Neurofilament Protein, Light Chain: An intermediate filament protein involved in cytoskeleton-mediated transport in axons and dendrites

Glutathione Synthetase: An enzyme that catalyzes GSH synthesis from gamma-glutamylcysteine and glycine in an ATP-dependent manner

Gamma-Glutamylcyclotransferase: An enzyme in the GSH metabolism that catalyzes the formation of 5-oxoproline from gamma-glutamyl dipeptides

Glutamate-Cysteine Ligase Catalytic Subunit: A rate-limiting enzyme in GSH synthesis

Gamma-Glutamyltransferase 1: An enzyme involved in the GSH catabolism by virtue of its role in the transfer of the glutamyl moiety of GSH to dipeptide acceptors

Glucose-6-Phosphate Dehydrogenase: Mediates the synthesis of nicotinamide–adenine dinucleotide phosphate- NADPH- to keep GSH in its reduced form

Superoxide Dismutase 1: An enzyme involved in the detoxification of superoxide free radicals

ATPase Na+/K+ Transporting Subunit Alpha 1: An integral membrane ion pump critically involved in the regulation of neuronal excitability by virtue of its role in establishing and maintaining the electrochemical gradients of sodium and potassium ions across the plasma membrane

Sarcoplasmic/endoplasmic reticulum (ER) Ca2+ transporting ATPase 2): An intracellular ion pump that sequesters free ionized calcium into the ER lumen

Ryanodine Receptor 1: A calcium ion channel that mediates release of Ca2+ from intracellular stores

Voltage Dependent Anion Channel 1: An ion channel in the mitochondrial outer membrane involved in mitochondrial calcium homeostasis

Glutamate Metabotropic Receptor 5: A membrane anchored G-protein coupled receptor for glutamate mediated signal transduction

Phospholipase C Gamma 1: A calcium ion binding protein that catalyzes the formation of second messengers molecules diacylglycerol and inositol 1,4,5-trisphosphate during receptor-mediated tyrosine kinase signal transduction

Autophagy Related 3: An E2 conjugating enzyme mediating cytoplasm to vacuole transport during autophagy, and also involved in mitochondrial homeostasis

Lysosomal Associated Membrane Protein 2: A factor in the chaperone-mediated autophagy and is required for the fusion of autophagosomes with lysosomes

Microtubule Associated Protein 1 Light Chain 3 Alpha (LC3): A factor involved in the formation of autophagosome

PTEN Induced Kinase 1: A serine/threonine protein kinase involved in mitochondrial quality control and mediating mitophagy of damaged mitochondria

Rubicon Autophagy Regulator: A negative regulator of endocytic trafficking and autophagy

Unc-51 Like Autophagy Activating Kinase 1: A serine/threonine protein kinase involved in the autophagosome formation

TANK Binding Kinase 1: A serine/threonine protein kinase phosphorylates several autophagy mediators and is involved in selective autophagy and inflammation
